# Genome-Wide Analyses of the Soybean F-Box Gene Family in Response to Salt Stress

**DOI:** 10.3390/ijms18040818

**Published:** 2017-04-12

**Authors:** Qi Jia, Zhi-Xia Xiao, Fuk-Ling Wong, Song Sun, Kang-Jing Liang, Hon-Ming Lam

**Affiliations:** 1 Key Laboratory of Ministry of Education for Genetics, Breeding and Multiple Utilization of Crops, College of Crop Science, Fujian Agriculture and Forestry University, Jinshan, Fuzhou 350002, China; jiaqi@fafu.edu.cn (Q.J.); sunsong07@163.com (S.S.); liangkj_2005@126.com (K.-J.L.); 2 School of Life Sciences and Center for Soybean Research of the Partner State Key Laboratory of Agrobiotechnology, The Chinese University of Hong Kong, Shatin, Hong Kong, China; obennoname@gmail.com (Z.-X.X.); wongfl@gmail.com (F.-L.W.)

**Keywords:** soybean, F-box gene family, expression patterns, salt stress

## Abstract

The F-box family is one of the largest gene families in plants that regulate diverse life processes, including salt responses. However, the knowledge of the soybean F-box genes and their roles in salt tolerance remains limited. Here, we conducted a genome-wide survey of the soybean F-box family, and their expression analysis in response to salinity via in silico analysis of online RNA-sequencing (RNA-seq) data and quantitative reverse-transcription polymerase chain reaction (qRT-PCR) to predict their potential functions. A total of 725 potential F-box proteins encoded by 509 genes were identified and classified into 9 subfamilies. The gene structures, conserved domains and chromosomal distributions were characterized. There are 76 pairs of duplicate genes identified, including genome-wide segmental and tandem duplication events, which lead to the expansion of the number of F-box genes. The in silico expression analysis showed that these genes would be involved in diverse developmental functions and play an important role in salt response. Our qRT-PCR analysis confirmed 12 salt-responding F-box genes. Overall, our results provide useful information on soybean F-box genes, especially their potential roles in salt tolerance.

## 1. Introduction

Organisms have developed multiple regulatory mechanisms to coordinate their life processes in response to internal or external stimuli. Among them, protein degradation via the ubiquitin/26S proteasome system (UPS) is an important post-translational regulatory mechanism, which is highly conserved in eukaryotes [1]. The UPS catalyzes the covalent attachment of multiple ubiquitins to the target substrate, which then causes the substrate to be recognized by the 26S proteasome for proteolysis. Ubiquitination includes sequential actions of three proteins: E1s (ubiquitin-activating enzymes), E2s (ubiquitin-conjugating enzymes) and E3s (ubiquitin protein ligases), of which E3s are the most diverse group, offering an extensive range of substrate selection [2]. One major and well characterized type of E3s is the SKP1-Cullin-F-box (SCF) complex made up of: suppressor of kinetochore protein 1 (SKP1)/Arabidopsis SKP1-like (ASK), Cullin 1 (CUL1)/cell division control protein 53 (CDC53), ring-box 1 (RBX1)/regulator of Cullins 1 (ROC1) and an F-box protein [3,4,5]. The first three subunits form a scaffold to assemble a diverse group of F-box proteins which confer the specificity for the target substrates to be degraded [5,6]. 

Numerous F-box proteins have been identified in eukaryotes in the past decades [5,6]. In plants, the F-box protein family is also one of the largest and most diverse gene families [7,8,9,10]. Around 694, 687, 359, 285, 972 and 517 F-box genes have been reported in Arabidopsis, rice, maize, chickpea, *Medicago truncatula* and apple, respectively [11,12,13,14,15,16]. F-box proteins contain at least one conserved F-box motif at its N-terminus, which interacts with SKP1 to form the SCF complex. Their C-termini are usually composed of one or several protein–protein interaction domains for specific substrate binding. Such a large number of F-box genes in plants implies that F-box proteins can recognize many different targets for UPS degradation, suggesting they might be involved in the regulation of many biological processes. Though a tremendous amount of F-box genes have been predicted in plants, only some of them have been characterized to show their roles in regulating a variety of life processes, including hormonal responses, lateral root formation, branching, senescence, light signaling, circadian rhythm, floral development, self-incompatibility, and responses to abiotic and biotic stresses [2,5,6,17]. 

The information on plant F-box genes was mostly obtained from the studies of Arabidopsis and rice, while the knowledge on soybean F-box genes (*GmFBXs*) is quite limited. To our knowledge, although hundreds of F-box genes have been predicted in soybean, only two, *GmCOI1* and *GmZTL3*, have been cloned and functionally characterized. *GmCOI1* shares a high-percentage homology with *AtCOI1* and is likely to be involved in the jasmonate pathway [18]. *GmZTL3* is a homolog of *AtZTL* and is involved in light signaling [19]. Hua et al. [7] made the phylogenic comparison of F-box genes in 18 plants, including the soybean, to show the expansion, evolutionary selection and functional correlation of F-box gene family. The results suggested that the diversification of F-box genes probably happened through genomic drift. Bellieny-Rabelo et al. [20] studied the impact of duplications on the F-box genes in legumes and found that *GmFBXs* evolved mainly through segmental duplications, consistent with the results by Hua et al. [7]. Nonetheless, the genome-wide analysis of *GmFBXs* is yet to be studied in depth. 

Cultivated soybean is a moderately salt-sensitive plant and high salinity could drastically reduce its yield [21]. In the past decades, though dozens of salt-tolerance genes have been identified and functionally characterized in the soybean [22,23,24], the detailed mechanisms of salt tolerance are still unknown. Fortunately, full genome sequencing of the soybean has been completed [25,26,27], supplying a valuable resource for the genome-wide analysis of gene families, including the salt-responsive homeodomain-leucine zipper (HD-Zip) transcription factor family [28], WRKY transcription factor family [24,29] and ankyrin repeats family [30]. In this study, we aimed to identify and characterize *GmFBXs* from a new version of the reference soybean genome (Wm82.a2.v1), with an emphasis in identifying F-box genes involved in salt stress responses, using in silico expression analysis with available RNA-sequencing (RNA-seq) data. Quantitative reverse-transcription polymerase chain reaction (qRT-PCR) was used to validate the differential expression of some target genes. These results provide useful information for further functional studies on *GmFBXs* and their possible roles in salt tolerance.

## 2. Results

### 2.1. Identification of F-Box Genes in Soybean

To identify possible members of the F-box protein-coding gene family in soybean, two strategies were used. First, the HMMER3.0 software [31] was employed to search against the soybean proteome database *Glycine max* Wm82.a2.v1 [32] using the hidden Markov model (HMM) profile (File S1) of F-box motifs (PF00646) downloaded from Pfam [33] as queries. A total of 708 transcripts from 503 genes encoding for F-box proteins were identified in the soybean genome (*E*-value cutoff 1.0). Meanwhile, a BLASTP search was also performed with an *E*-value cut off 1.0 to identify 875 transcripts encoded by 602 genes. By combining the results from the two searches, the redundant sequences with the same accessions were removed and the non-redundant sequences were then confirmed using the InterPro database [34]. Finally, 725 potential F-box-containing protein transcripts encoded by 509 genes were identified, which were named based on the chromosomal distribution of the corresponding genes (Table S1). The results revealed that the molecular weight, isoelectric point and putative subcellular localization of these proteins varied widely. Alternative transcripts from the same gene locus were designated under the same gene name, such as *GmFBX3.1* and *GmFBX3.2*. Alternative splicing, which enables a single coding sequence to produce more than one messenger RNA (mRNA) product, is an important mechanism for regulating proteomic diversity and gene expression [35]. One hundred and twenty-one of 509 F-box genes (23.8%) contained two to ten alternative structures due to alternative splicing (Table S1), suggesting various transcript isoforms generated from the same gene locus could have different functions in different cell types, developmental stages and stress responses.

### 2.2. Analysis of Conserved Domains and Classification of Soybean F-Box Proteins

**Figure 1:**
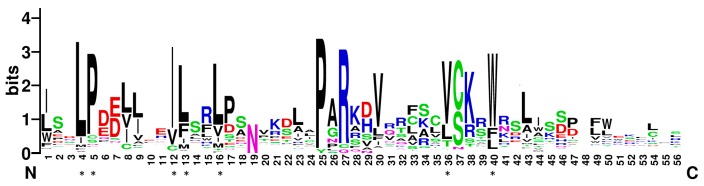
WebLogo based on the alignments of the F-box motifs from 509 F-box proteins in soybean. The overall height of each stack represents the sequence conservation at the given position of the F-box motif, whereas the height of the letters within each stack indicates the relative frequency of the corresponding amino acid. The bit score represents the information content for each position. Asterisks indicate the conserved residues.

F-box proteins usually contain an approximately 40–60 amino-acid F-box motif at the N-terminus [10]. In this study, most of the soybean F-box proteins contain only one F-box motif. Only GmFBX505 contains two. To characterize the conserved residues in soybean F-box motifs, the sequences of the F-box domains from the above 509 genes were aligned (File S2) and a Weblogo [36] was generated (Figure 1). The highly conserved residues in soybean F-box motifs were Ile-12 (the Ile residues at position 48) (75.8%), Pro-5 (72.1%), Leu-4 (71.7%) and Trp-40 (70.7%), followed by Leu-16 (68.6%), Val-36 (68.2%) and Leu-13 (65.0%). When aligned with the conserved residues of F-box motifs in Arabidopsis, maize, apple, *Medicago trucatula* and the conserved SKP1-binding residues of the human SKP2 [11,13,15,16], the soybean version shared most of the conserved residues with these other F-box motifs, suggesting these conserved residues could be the binding sites for SKP proteins to form the SCF complex in soybean as well.

**Figure 2:**
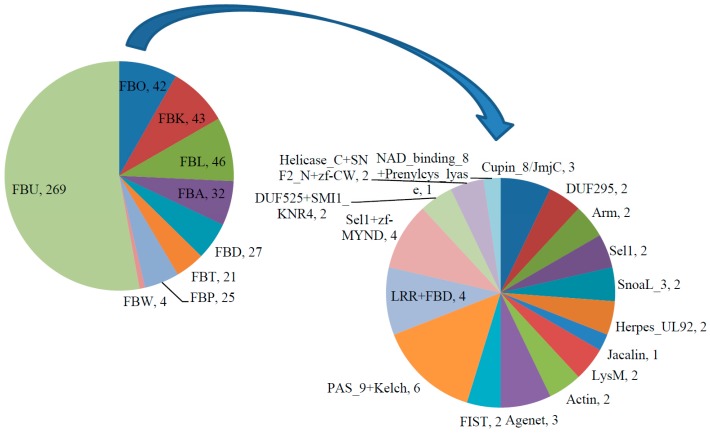
Classification of F-box proteins in soybean based on C-terminal domains. The F-box proteins were classified into nine subfamilies according to their C-terminal domains. FBU: F-box proteins with unknown C-terminal domains; FBL: those with C-terminal leucine-rich repeats (LRR) domains; FBK: those with C-terminal Kelch domains; FBA: those with C-terminal F-box-associated domains; FBD: those with C-terminal F-box domains; FBT: those with C-terminal tubby (TUB) domains; FBP: those with phloem protein 2 (PP2) domains; FBW: those with WD40 domains; FBO: those with other known domains or with more than one C-terminal domains. The composition of the FBO subfamily is further expanded and represented by the second pie chart. The number after the comma represents the number of F-box proteins in that group. NAD: nicotinamide adenine dinucleotide; DUF295: domain of unknown functions 295; FIST: F-box and intracellular signal transduction; PAS_9: Per-ARNT-Sim 9; LysM: Lys motif; Arm: armadillo repeats; Herpes_UL: herpes virus long unique region; zf-MYND: MYND type zinc finger; MYND: myeloid, Nervy, and DEAF-1, deformed epidermal autoregulatory factor 1; SnoaL: nogalonic acid methyl ester cyclase from *S. nogalater.* SNF2_N: SNF2 family N-terminal domain; Prenylcys_lyase: prenylcysteine lyase.

In addition to the N-terminal F-box motif, most F-box proteins contain a diverse array of functional domains at the C-terminus, which have been proven or predicted to be protein–protein interaction domains for recognizing various substrates of the SCF complex [10]. Using the SMART, InterPro and Pfam databases [32,37,38], the C-terminal regions of soybean F-box proteins were investigated. Like the F-box genes in other plants [8,10,11,13,14,15,16,39], the search results showed that 269 of the predicted soybean F-box proteins did not contain any known domain at the C-terminus and the others (238) contained one or several known functional domains including leucine-rich repeats (LRR), Kelch repeats, F-box associated domain (FBA), F-box domain (FBD), tubby (TUB), phloem protein 2 (PP2), WD40 repeats, Sel1 repeats, zinc finger (ZF), Per-ARNT-Sim (PAS)/PAC domain, armadillo (ARM)/beta-catenin-like repeats, Actin, Agenet, F-box and intracellular signal transduction (FIST) domain, Cupin_8/Jumoji C (JmjC) domain, domain of unknown functions 295 (DUF295), domain of unknown functions 525 (DUF525), SMI1_KNR 4 domain, Helicases_C, SNF2 family N-terminal domain (SNF2_N), SnoaL_3, Herpesvirus UL92 (Herpes_UL92), Jacalin, Lys motif (LysM), NAD_binding_8, and prenylcysteine lyase (Prenylcys_lyase). Based on these results, *GmFBXs* were classified into 9 subfamilies (Figure 2). Two hundred and sixty-nine soybean F-box proteins without any known domain at the C-terminus were classified in the F-box proteins with unknown C-terminal domains (FBU) subfamily, those with C-terminal leucine-rich repeats (LRR) domains in the (FBL) subfamily (46), those with C-terminal Kelch domains in the; FBK subfamily (43), those with C-terminal F-box-associated domains in the FBA subfamily (32), those with C-terminal F-box domains; in the FBD subfamily (27), those with C-terminal tubby (TUB) domains in the FBT subfamily (21), those with PP2 in the FBP subfamily (25), those with WD40 in the FBW subfamily (4), and those with other known domains or more than one known domain in the FBO subfamily (424). As a further step, the C-terminal unknown domains were analyzed using the MEME (Multiple EM for Motif Elicitation) program [40]. Thirty-nine putative conserved motifs with statistical significance (*E*-value less than *E*-100) were identified. Each of these motifs was more than 15 amino acids in length and was found to be conserved in at least four putative F-box genes (Table S2). Among them, twelve of these motifs were conserved in more than 20 *GmFBXs*. 

### 2.3. Phylogenetic Analyses Based on Gene Structures

**Figure 3:**
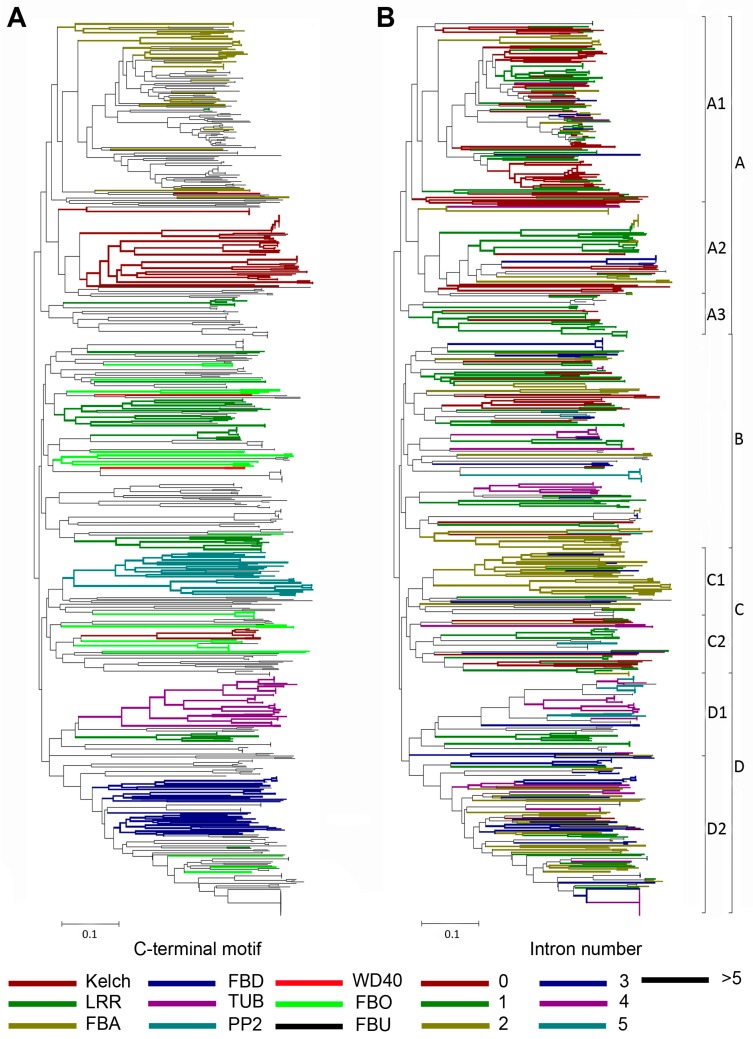
Phylogenetic analysis of the F-box protein in soybean. The unrooted neighbor joining (NJ) tree was constructed based on a complete alignment of the F-box motifs from the 725 predicted F-box proteins using the *p*-distance method and a bootstrap value of 1000 with the program MEGA6 [41]. Scale bar represented the branch length (equivalent to 0.1 amino acid substitution per residue). Individual members of the tree were color-coded by the C-terminal conserved domains (**A**), or the number of introns (**B**) within the respective genes. Clades were divided into 4 groups (A–D) and 9 subgroups (A1–A3, B, C1–C2, D1–D2), marked on the right. The expanded versions are available as Files S3 and S4.

Previous phylogenetic studies on plant F-box proteins using only the F-box motifs for alignment showed that the F-box proteins with the same C-terminal domains tended to cluster in the same clades of the phylogenetic tree, and the intron–exon organization was conserved within the same clade [10,11,12,13,15,39]. Here, we aligned the 725 soybean F-box proteins using the F-box motifs, to determine the phylogenetic relationships among them. In the case of GmFBX505, which contains two F-box motifs, the sequence of the N-terminal motif was employed in the alignment. A phylogenetic tree was created using the neighbor-joining method (Figure 3). Based on the node statistics and the branch lengths, the 725 soybean F-box proteins were tentatively assigned to four groups designated from A–D, which were further divided into eight subgroups. Group A was divided into 3 subgroups, Group C into 2, and Group D into 2. The color-coded phylogenetic tree based on the C-terminal conserved domains showed that the F-box proteins with identical domains also cluster together using the traditional F-box domain alignments, supporting our way of classification based on the C-terminal domains in soybean (Figure 3A). 

Since the exon–intron organization could be used to investigate the evolutionary relationships of multigene families [8,42,43], we analyzed the exon–intron structures in the coding sequences of *GmFBXs*. A total of 42.95% of the introns were less than 200 bp and the largest intron was 10,545 bp (*GmFBX229.2*). The number of introns within the open reading frame (ORF) of each F-box gene ranged from 0 to 16. Four hundred and sixty-five (64.14%) of the 725 distinct transcripts from the annotated F-box genes in soybean contained fewer than 3 introns, whereas only 8.44% (61) contained more than 5 introns. When the phylogenetic tree was color-coded to reflect the intron number of each F-box gene, it showed that in the same subclade the genes with similar exon–intron structures generally clustered together (Figure 3B). Remarkably, the genes within the same subgroup shared the same intron pattern and a similar type of alternative splicing. For instance, in the D2 subgroup, the alternative transcripts from *GmFBX231* and *GmFBX230* were predicted to contain 2 or 3 introns, while *GmFBX226*, *GmFBX227* and *GmFBX228* were predicted to contain 3 or 4 introns. Therefore, the exon–intron architecture analyses also partly support our phylogenetic maps based on the F-box motifs and the C-terminal conserved motifs.

### 2.4. Chromosomal Distribution and Gene Duplication

**Figure 4:**
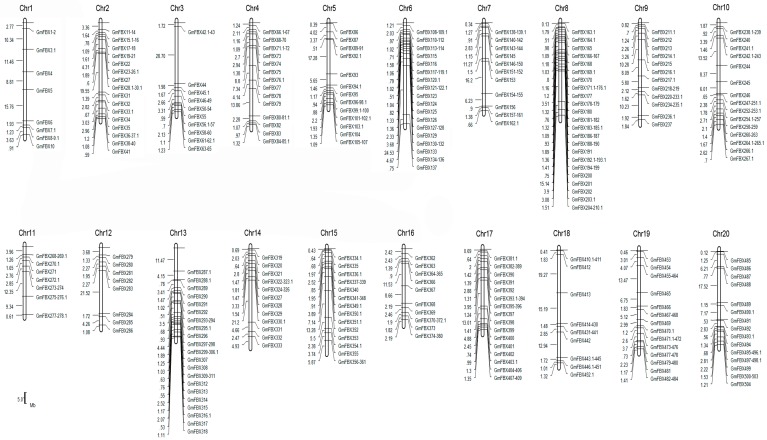
Chromosomal locations of *GmFBXs* in soybean. The chromosomes are drawn to scale and the chromosome numbers are indicated at the top of each bar. Chromosomal distances are given in megabases (Mb). The scale bar represents 5 Mb. The gene names are shown on the right side of each chromosome corresponding to the position of each gene.

**Figure 5:**
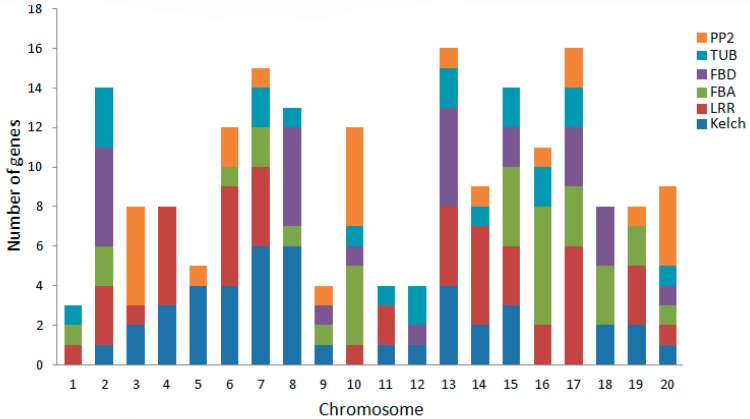
Chromosomal distributions of *GmFBXs* with different conserved C-terminal domain types in soybean. The different C-terminal domains (Kelch, LRR, FBA, FBD, TUB, PP2) are represented by the corresponding colors. LRR: leucine-rich repeat; FBA: F-box associated domain; FBD: F-box domain; TUB: tubby; PP2: phloem protein 2.

The large number of F-box genes in soybean reflects the gene duplication events that happened during the evolution of soybean. It has been recognized that segmental duplication and tandem duplication are the main causes of gene family expansion, thereby establishing the diversification of gene functions in plant evolution [37]. To understand the mechanism of soybean F-box gene evolution and the diverse functions of the F-box genes in response to salt stress, the locations of the F-box genes on the soybean chromosomes were investigated with respect to soybean genome duplication events. A chromosome map was constructed to determine the chromosomal distribution of the F-box protein-encoding genes in soybean (Figure 4). Among all *GmFBXs*, 504 were mapped to the soybean chromosomes and the others were found in the scaffolds. The results reveal that *GmFBX*s are found in clusters on all the chromosomes and most of them are distributed on chromosome arms. Chromosome 8 contains the highest number of *GmFBX*s (48), followed by chromosome 18 (43), whereas chromosome 12 contains the fewest *GmFBX*s (8). Clustering of *GmFBX*s is particularly prominent on chromosomes 8, 18 and 19. When the positions of the F-box genes with different C-terminal domains are indicated on the chromosomal map (Figure 5), it is evident that the chromosomal distribution of *GmFBXs* is strongly tied to their C-terminal domains. For example, most *GmFBX*s with Kelch domains are located on chromosomes 5, 6, 7, 8 and 13; those with LRR domains on chromosomes 4, 6, 7, 13, 14 and 17; those with FBA domains on chromosomes 10, 15 and 16; those with FBD domains on chromosome 2, 8 and 13; and those with PP2 domains on chromosomes 3, 10 and 20. Furthermore, in some cases, *GmFBXs* containing the same C-terminal domain were clustered on the same chromosome. For example, *GmFBX377-GmFBX380* which encode F-box proteins with an FBA domain, are located within an 18 kb segment on chromosome 16, suggesting that a tandem duplication event occurred in the evolution of *GmFBXs*. 

Based on the phylogenetic relationship, approximately 76 pairs of paralogous genes were identified, as supported by strong bootstrap value (>90%) and high level of sequence similarity. However, only 12 pairs were found to be tandem duplicates. This suggests that segmental duplications probably played a bigger role in the expansion of *GmFBXs*, which is consistent with a recent report on the F-box family in legumes [20]. To date the duplication times and study the selection pressures on amino-acid substitutions among the paralogous genes, the non-synonymous (*K*) value, the synonymous (*K*s) value, and the ratios of *K*a versus *K*s mutations (*K*a/*K*s) were calculated (Table S3). The approximate dates of duplication events were calculated using *K*s. The results showed that the divergence periods were from 1.01 Mya (a million years ago) to 185.94 Mya, with an average of 14.61 Mya for the segmentally duplicated genes, and from 0.30 Mya to 26.13 Mya with an average of 5.18 Mya for the tandemly duplicated genes. The *K*a/*K*s ratios of duplicated gene pairs varied from 0 to 2.13 with an average of 0.34. The values of *K*a/*K*s ratio were less than or nearly equal to 1 for most gene pairs, suggesting that these gene pairs were mainly under purifying selection pressure. For the duplicated gene pairs of *GmFBX134/135* and *GmFBX451/505*, the *K*a/*K*s ratios were more than 1, indicating the positive selection.

### 2.5. In Silico Expression Patterns of GmFBXs

**Figure 6:**
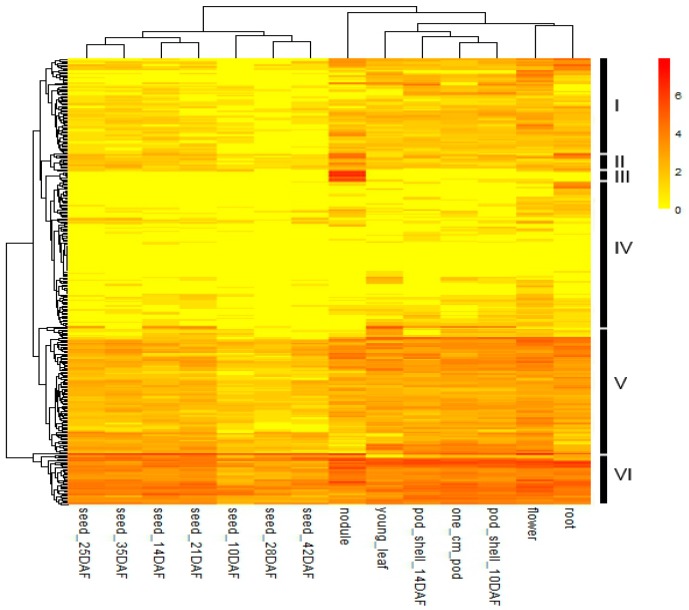
Heat map exhibiting the expression profiles of *GmFBXs* in 14 tissues. The heat map was generated using the RNA-sequencing data downloaded from the online dataset Soybase [45]. The genes with Reads/Kb/Million (RPKM) values of 0 in all 14 tissues were not included. The normalized RPKM values of the expressed genes were log2-transformed. According to the expression pattern, F-box genes were clustered into six groups (I–VI). The color scale is shown on the right. DAF: days after flowering.

Gene expression patterns can provide crucial indications for gene functions. Some high-throughput next-generation sequencing analyses on gene expression have been performed on various soybean tissues [38,44]. To investigate the tissue-specific expressions of *GmFBXs*, the publicly available RNA-seq data were downloaded from Soybase [45]. It contains samples from 14 tissue types. The transcript patterns of 375 F-box genes were retrieved in terms of normalized Reads/Kb/Million (RPKM) values, and among them 317 genes were expressed in at least one of the 14 tissues. Those F-box genes which were missing in the dataset could be either pseudogenes or were only expressed under particular conditions or at specific developmental stages. A heat map of the differential transcript abundance of these 317 F-box genes in the 14 tissue types shows that most F-box genes have broad expression patterns in soybean (Figure 6). Many F-box genes (194 out of 317) were constitutively expressed in all 14 tissue types. Among them, 18 F-box genes were highly expressed, with a normalized RPKM value of higher than 4. F-box genes could be involved in multiple developmental processes in soybean. According to the hierarchical clustering tree based on their specific expression patterns in different tissues, *GmFBXs* could be divided into six groups (I–VI) (Figure 6). The expression levels of the Group I genes were lower during the seed development stage compared to the others. The genes of the Group II and III have a higher expression level in nodules. Interestingly, the 8 F-box genes of Group III were highly expressed particularly in nodules, suggesting their putative roles in nodulation. Compared with the other groups, the F-box genes in Group IV shared a low expression level and those in Group V showed a medium expression level throughout the entire plant, whereas the expression levels of those in Group VI were relatively high in most of the examined tissues. 

### 2.6. Expression Profiles of GmFBXs in Response to Salt Stress

To investigate the potential functions of *GmFBXs* in response to salt stress, we made use of the publicly available RNA-seq data (GSE57252, BioProject ID number: PRJNA246058 [28]). The normalized Fragments/Kb/Million (FPKM) values of 344 F-box genes were obtained from the salt-stress transcriptome data. The detailed transcriptome profile is shown as a heat map (File S5). A total of 51 F-box genes were considered as differentially expressed genes with 2 or more fold change with the false discovery rate (FDR) value of less than 0.05 under at least one of the three time points under salt stress. Among them, 34 genes were up-regulated and 17 down-regulated, indicating F-box genes would be involved in the response to salt stress in soybean.

**Figure 7:**
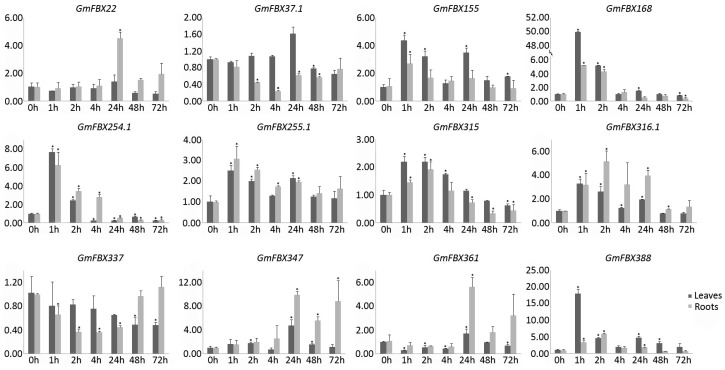
Relative expression levels of 12 *GmFBXs* in response to salt stress. RNA was extracted from the leaves and roots of soybean at 0 h, 1 h, 2 h, 4 h, 24 h, 48 h and 72 h after a 0.9% NaCl treatment. The quantitative reverse-transcription polymerase chain reaction (qRT-PCR) data were normalized with *GmELF1b* and the gene expression levels relative to those at 0 h were shown. The error bars indicate standard deviations (*t*-test, * *p* < 0.05).

To validate these findings and further determine the involvement of *GmFBXs* in response to salt stress, quantitative reverse-transcription PCR (qRT-PCR) was performed to analyze the expression profiles of the F-box genes under salt stress. Twelve differentially expressed genes were selected. The plants were treated with 0.9% NaCl for different lengths of time (0 h, 1 h, 2 h, 4 h, 24 h, 48 h and 72 h). The roots and leaves of the treated plants were collected separately. The expression profiles detected by qRT-PCR were generally consistent with those from the RNA-seq data for 9 of the 12 genes (Figure 7). The expression levels of *GmFBX168*, *GmFBX254.1*, *GmFBX315*, *GmFBX316.1* were induced significantly in roots at the early stages after salt treatment, whereas those of *GmFBX37.1* and *GmFBX337* were reduced. The expression of *GmFBX22*, *GmFBX347* and *GmFBX361* remained at a similar level in roots at the early stages under salt stress. Interestingly, they were induced significantly in roots at the later stages. Besides, the qRT-PCR results showed that the expression of *GmFBX155*, *GmFBX255.1* and *GmFBX388* were induced significantly in roots at the early stages of salt treatment, which is different from the RNA-seq data. We also analyzed the expression levels of the 12 genes in the aerial part. It seemed that most of them have a similar trend of changes in leaves as those in roots under salt stress. Some were significantly changed in both leaves and roots, such as *GmFBX168* and *GmFBX316.1*, indicating that they might take roles in the response to salinity in the whole plants. Some were changed more specifically only in roots or in leaves. For instance, the expression levels of *GmFBX337* were reduced only in roots under salt stress. They probably functioned in a specific part in the response to salt stress. In summary, the results of qRT-PCR also suggested that the *GmFBXs* would be involved in the response to salt stress.

## 3. Discussion

As F-box genes form a large gene family in plants and they were reported to be involved in multiple pathways, it is logical to speculate that they play important roles in a series of biological processes throughout the plant life cycle [6]. However, the functions of most plant F-box genes remain unknown, especially in soybean. In this study, 509 F-box genes were identified in the new version of the soybean genome database Wm82.a2.v1. Our list of *GmFBXs* is generally consistent with the former works [20] and is renewed according to the renewal of soybean genome database. They were classified into subfamilies based on gene structures, conserved motifs, phylogenetic relationships and chromosomal distributions. Extensive analyses of their expression patterns were also performed in different tissues at different developmental stages and under salt stress in silico and validated by qRT-PCR.

Conserved domain analysis revealed that the conserved residues of F-box motifs in *GmFBXs* were similar to those in other plants and the human SKP2. Therefore, the universal formation of the SCF complex could also occur in soybean. Besides, most of the known C-terminal functional domains in soybean have also been found in other plants, suggesting evolutionary gene conservation and similar roles of F-box proteins across different plants. According to different types of the C-terminal domain, soybean *GmFBXs* can be classified into nine subfamilies, among which the most abundant F-box proteins belong to the FBU subfamily, containing unknown C-terminal domains. Using MEME online tools, we identified 39 putative conserved domains. The BLAST searches of these unknown motifs revealed that most of them also exist in other plants, while a few are Fabaceae family- or soybean-specific. Like other known C-terminal conserved motifs, these putative novel motifs may also function in the interaction between F-box proteins and their substrates. Since we know little about the plant F-box genes, it is worth investigating the roles of these putative conserved domains with respect to their specific substrates and the cellular functions they may regulate. Those containing legume- or soybean-specific motifs may carry functions related to legume-specific cellular processes. 

The fact that the criterion of F-box gene classification used here, based on C-terminal substrate-interacting domains, re-affirms the previous classification based on N-terminal F-box motifs suggests that the N-terminal F-box domains are likely to have co-evolved with the C-terminal domains, similar to other plants [11,12,13,14,15]. Moreover, the phylogenetic tree generated by the F-box motifs was partly in agreement with the results of the gene structure analysis, indicating that this tree represented the phylogenetic relationship of *GmFBXs* accurately. However, there was one special characteristic of *GmFBXs* that was different from other plants. The intron-less F-box genes in soybean (15.58%) are much less common than in other plants reported thus far. Intron-less F-box genes have the highest percentage in Arabidopsis, rice, maize, chickpea and *Populus* [8,11,13,14,39]. For example, 45% of the F-box genes were predicted to be intron-free in Arabidopsis [11], 40.76% in rice [39], and more than 40% in maize [13]. This may indicate that *GmFBXs* evolved in a mode distinct from other plant species. Overlaying the phylogenetic analysis with the chromosomal distribution of F-box genes revealed that segmental duplications played a critical role in the gene expansion in soybean, consistent with other reports on duplication events in soybean [20]. 

The biochemical characteristics and subcellular localizations of soybean F-box proteins vary widely. Most of the soybean F-box proteins were predicted to be in the cytoplasm, nucleus and organelles. A few were predicted to be in the plasma membrane, extracellular space and vacuole. A large fraction of soybean F-box proteins were predicted to have multiple subcellular localizations. A report showed that most of the 17 F-box proteins tested were localized mainly in intracellular compartments and a single F-box protein could form multiple SCF complexes in Arabidopsis [46]. All these demonstrate that F-box proteins have diverse roles in regulating the biological processes in plants.

Expression analyses could provide insight into the potential functions of genes. RNA-seq is a powerful tool for investigating the transcription patterns of certain genes using high-throughput sequencing technology. Here we used publicly available RNA-seq data to study the expression profiles of *GmFBXs* in different tissues at various developmental stages and the expression profiles in roots under salt stress. Though most detected F-box genes were expressed broadly under normal growth conditions, many F-box genes showed specific differential expression patterns temporally and spatially, indicating that these *GmFBXs* would play diverse functions in development. 

To investigate the functional divergence, we also compared the expression levels of the duplicate genes. Among the 76 pairs of duplicate genes, the expression of 22 pairs was not detected in any tissues under normal growth condition. Either these genes play a highly specific role in response to a particular growth condition not tested here or they are simply pseudogenes. More than half of the duplicate gene pairs with detected expression levels showed a similar expression pattern between the duplicates, suggesting that they might have redundant roles. A few duplicated gene pairs seemed to have different expression patterns. For example, *GmFBX122* had much higher expression in all the detected tissues than its paralog *GmFBX330.1*. *GmFBX146* was detected in most tissues and its paralog *GmFBX148* was only detected in seeds, suggesting that they might have different functions. From the RNA-seq data of salt treatment, the expression patterns of most duplicated gene pairs were similar, but some pairs were slightly different. The expression of *GmFBX128* was decreased significantly at 1 h, increased at 6 h and decrease again at 12 h under salt treatment, whereas the expression of *GmFBX80* was slightly decreased at 1 h, and increased at 6 h and 12 h under salt treatment. The promoter analysis via PlantCARE online tool [47] showed that there are always differences in the *cis*-elements between the duplicates. All these indicated that sub- and neo-functionalization could occur during the duplication process of F-box genes in soybean. 

Besides, a list of salt-responding genes was explored in this study. A total of 51 *GmFBXs* were identified as differentially expressed genes in response to salt stress from the RNA-seq data (BioProject ID number: PRJNA246058). Through combining our preliminary transcriptome data of salt treatments, twelve genes were selected for further analyzing their expression levels after salt treatments via qRT-PCR. According to the online RNA-seq data of dehydration treatments [28], some genes had different expression patterns between dehydration stresses and salt stresses. Among the 12 selected *GmFBX* genes, the expression patterns of five genes (*GmFBX254.1*, *GmFBX255.1*, *GmFBX313*, *GmFBX314* and *GmFBX337*) were different, suggesting that they might play roles, particularly in the salt response. Until now, most of the salt-responding *GmFBXs* have not been characterized. Further functional analysis are needed to unveil their functions and mechanisms of salt responses in soybean. 

## 4. Materials and Methods

### 4.1. Database Search for F-Box Proteins in Soybean

The HMM profile (File S1) of F-box domain (PF00646) containing 463 seed sequences was downloaded from Pfam [33]. To identify the F-box genes in soybean, two strategies were used here. One was to employ HMMER3.0 software [31] to search against the latest soybean (*Glycine max*) whole proteome database (Wm82.a2.v1) on Phytozome 10 [32]. The *E*-value cutoff was set at 1.0. In addition, The BLASTP search was also performed with an *E*-value cutoff 1.0. The redundant sequences with the same accessions were removed from the dataset. The retrieved sequences of the candidate proteins were then submitted to the InterPro database to ensure the presence of F-box domains [34].

### 4.2. Sequence Analysis of Conserved Domains

The conserved domains in F-box proteins were identified using SMART) [48], InterPro, and Pfam, with an *E*-value cutoff of 1.0. The sequences of all the F-box motifs were aligned using CLUSTALX2.1 [49]. The sequence logo was generated using the online program WebLogo [36]. All the sequences of soybean F-box proteins with C-terminal unknown domains were sent to the MEME website to identify the unknown conserved domains [40]. The parameters were set as follows: zero or one per sequence for distribution of motif occurrences, 50 for the maximum number of motifs, and 6–50 amino acids for optimum motif width. All the other options were set at default. 

### 4.3. Phylogenetic Analysis, Gene Structure Analysis and In Silico Prediction of Subcellular Localizations

Multiple sequences of the F-box domains from all the soybean F-box proteins were aligned using CLUSTALX2.1 [49]. An unrooted neighbor-joining tree of the alignment was constructed using the *p*-distance method with gaps treated by pairwise deletion and a bootstrap value of 1000 replicates by MEGA6 [41]. The chromosomal locations and exon-intron organizations of *GmFBXs* were obtained from Phytozome. Molecular weights and theoretical isoelectric points (pIs) of the soybean F-box proteins were computed using Compute pI/Mw tool on the ExPASy server [50]. Protein subcellular localizations were predicted using YLoc [51].

### 4.4. Chromosomal Distribution and Gene Duplication Analyses

The physical locations of all *GmFBXs* were obtained from the Phytozome database and visualized using Mapdraw [52]. A neighbor-joining tree was constructed with the full-length protein sequences encoded by all the F-box genes. Duplicated genes were identified in the terminal nodes of the phylogenetic tree with strong bootstrap values (>90%) and high sequence similarities. The criteria of sequence similarity used for determining the duplicated genes are that the length of the aligned sequences covers more than 80% of the longer gene and the identity is more than 70% [53,54]. Duplicated genes located within 20 loci on the same chromosome were considered as tandem duplicates and duplicated genes on different chromosomes were designated as segmental duplicates [55]. Pairwise alignments of the paralogous nucleotide sequences were constructed using ClustalX2.1. The program DnaSP v5 was used to estimate synonymous substitution rate (*K*s) and non-synonymous substitution rate (*K*a) [56]. The ratio of *K*a/*K*s was calculated to detect the mode of selection. For each gene pair, the *K*s value was translated into the approximate date of the duplication event (*T* = *K*s/2*λ*), assuming the mean value of clock-like rates (*λ*) of synonymous substitution is 6.1 × 10^−9^ [57,58]. One thousand five hundred-bp genomic DNA sequences upstream from the transcriptional start site of the duplicates were downloaded via Phytozome 10 and sent to the PlantCARE website [47] to analyze the *cis*-elements in the promoters. 

### 4.5. Digital Expression Analyses of GmFBXs

The normalized RPKM values from the transcriptome data of fourteen tissues were downloaded at Soybase [38,45] to investigate the tissue-specific expression profiles of *GmFBXs*. The 14 tissues are as follows: young leaf, flower, 1 cm pod, pod shell 10 days after flowering (DAF), pod shell 14 DAF, seed 10 DAF, seed 14 DAF, seed 21 DAF, seed 25 DAF, seed 28 DAF, seed 35 DAF, seed 42 DAF, root, and nodule. The values of normalized RPKM were log2-transformed and displayed in a heat map. The heat map was generated in R using the pheatmap function in the pheatmap CRAN (Comprehesive R Archive Network) library [59]. 

To determine the involvement of *GmFBXs* in response to salt stress, the publically available transcriptome data of soybean under salt treatments were downloaded from the NCBI Gene Expression Omnibus (GEO) database (GSE57252) [28]. The Illumina sequencing data were obtained from the soybean roots treated with 100 mM of NaCl at three time points (1 h, 6 h and 12 h), or the roots without salt treatment (control). The normalized RPKM values of the samples under salt treatments were compared to the normalized RPKM values of the samples without salt treatment. The ratios were log2-transformed for the heat map construction. Differentially expressed F-box genes under salt stress were selected through fold-change and *p* value with Benjamini Hochberg false discovery rate (FDR) multiple testing correction. The threshold was more than 2 for the fold-change and less than 0.05 for the *p*-value. Besides, the residual variance quotients of the samples are less than 20 [28].

### 4.6. Salt Treatments and Gene Expression Analyses

The salt treatments on soybeans were performed as previously described [22]. The seeds of the cultivated soybean accession C08 were germinated in vermiculite saturated with water. One-week-old seedlings were transferred to a hydroponic system with half-strength Hoagland’s nutrient solution. After the opening of the first trifoliate, the seedlings were treated with half-strength Hoagland’s solution containing 0.9% NaCl for different lengths of time (0 h, 1 h, 2 h, 4 h, 24 h, 48 h and 72 h). The roots and leaves of the treated plants were harvested separately and frozen in liquid nitrogen for total RNA extraction. Three individual plants of the same treatment were pooled as one sample and two independent biological replicates were performed for gene expression analyses.

Quantitative reverse-transcription PCR was performed using the PrimerScript one step RT-PCR kit (TaKaRa Biotechnology Co., Ltd., Dalian, China) according to the manufacturer's instructions. The primers for qRT-PCR are listed in Table S4. Relative gene expression was calculated using the 2^−ΔΔ*C*t^ method [60]. The data were normalized to the reference gene *ELF1b* [61]. The qRT-PCR reactions were performed with at least three replicates. 

## Supplementary Materials

Supplementary materials can be found at www.mdpi.com/1422-0067/18/4/818/s1. Supplementary Files S1–S5: File S1. The seed sequences of the F-box domain (PF00646) downloaded from Pfam; File S2. Sequence alignment of soybean F-box domains; File S3. Phylogenetic tree of the soybean F-box proteins color-coded by the C-terminal domains. This is the more detailed version of Figure 3a; File S4. Phylogenetic tree of the soybean F-box proteins color-coded by the number of introns. This is the more detailed version of Figure 3b; File S5. Heat map exhibiting the expression profiles of soybean F-box genes under salt tress. The expression levels of each gene under salt treatments were compared to those at 0h. The relative magnitude of fold changes in log2 values were presented by the intensities of the color and the color scale is shown on the right. Na1hr, Na6hr and Na12hr represent data from soybean roots at 1h, 6h and 12h after salt treatments, respectively. Supplementary Tables S1–S4: Table S1. List of soybean F-box proteins; Table S2. Putative motifs predicted in soybean F-box proteins of FBX subfamily by MEME with an *E-*value less than *E*-100; Table S3. *K*a/*K*s analysis and estimated divergence time for the *GmFBX* duplicated genes; Table S4. List of primers for qRT-PCR.

## References

[1] Smalle J., Vierstra R.D. (2004). The ubiquitin 26S proteasome proteolytic pathway. Annu. Rev. Plant Biol..

[2] Sadanandom A., Bailey M., Ewan R., Lee J., Nelis S. (2012). The ubiquitin-proteasome system: Central modifier of plant signalling. New Phytol..

[3] Vierstra R.D. (2009). The ubiquitin-26S proteasome system at the nexus of plant biology. Nat. Rev. Mol. Cell Biol..

[4] Chen L., Hellmann H. (2013). Plant E3 Ligases: Flexible Enzymes in a Sessile World. Mol. Plant.

[5] Somers D.E., Fujiwara S. (2009). Thinking outside the F-box: Novel ligands for novel receptors. Trends Plant Sci..

[6] Lechner E., Achard P., Vansiri A., Potuschak T., Genschik P. (2006). F-box proteins everywhere. Curr. Opin. Plant Biol..

[7] Hua Z., Zou C., Shiu S.-H., Vierstra R.D. (2011). Phylogenetic comparison of F-Box (FBX) gene superfamily within the plant kingdom reveals divergent evolutionary histories indicative of genomic drift. PLoS ONE.

[8] Yang X., Kalluri U.C., Jawdy S., Gunter L.E., Yin T., Tschaplinski T.J., Weston D.J., Ranjan P., Tuskan G.A. (2008). The F-box gene family is expanded in herbaceous annual plants relative to woody perennial plants. Plant Physiol..

[9] Navarro-Quezada A., Schumann N., Quint M. (2013). Plant F-box protein evolution is determined by lineage-specific timing of major gene family expansion waves. PLoS ONE.

[10] Xu G., Ma H., Nei M., Kong H. (2009). Evolution of F-box genes in plants: Different modes of sequence divergence and their relationships with functional diversification. Proc. Natl. Acad. Sci. USA.

[11] Gagne J.M., Downes B.P., Shiu S.-H., Durski A.M., Vierstra R.D. (2002). The F-box subunit of the SCF E3 complex is encoded by a diverse superfamily of genes in Arabidopsis. Proc. Natl. Acad. Sci. USA.

[12] Kuroda H., Takahashi N., Shimada H., Seki M., Shinozaki K., Matsui M. (2002). Classification and expression analysis of Arabidopsis F-box-containing protein genes. Plant Cell Physiol..

[13] Jia F., Wu B., Li H., Huang J., Zheng C. (2013). Genome-wide identification and characterisation of F-box family in maize. Mol. Genet. Genom..

[14] Gupta S., Garg V., Kant C., Bhatia S. (2015). Genome-wide survey and expression analysis of F-box genes in chickpea. BMC Genom..

[15] Song J.B., Wang Y.X., Li H.B., Li B.W., Zhou Z.S., Gao S., Yang Z.M. (2015). The F-box family genes as key elements in response to salt, heavy mental, and drought stresses in *Medicago truncatula*. Funct. Integr. Genom..

[16] Cui H.-R., Zhang Z.-R., Lv W., Xu J.-N., Wang X.-Y. (2015). Genome-wide characterization and analysis of F-box protein-encoding genes in the *Malus domestica* genome. Mol. Genet. Genom..

[17] Stone S.L. (2014). The role of ubiquitin and the 26S proteasome in plant abiotic stress signaling. Front. Plant Sci..

[18] Wang Z., Dai L., Jiang Z., Peng W., Zhang L., Wang G., Xie D. (2005). GmCOI1, a soybean F-box protein gene, shows ability to mediate jasmonate-regulated plant defense and fertility in Arabidopsis. Mol. Plant Microbe Interact..

[19] Xue Z.-G., Zhang X.-M., Lei C.-F., Chen X.-J., Fu Y.-F. (2012). Molecular cloning and functional analysis of one ZEITLUPE homolog GmZTL3 in soybean. Mol. Biol. Rep..

[20] Bellieny-Rabelo D., Oliveira A.E.A., Venancio T.M. (2013). Impact of whole-genome and tandem duplications in the expansion and functional diversification of the F-box family in legumes (Fabaceae). PLoS ONE.

[21] Phang T.-H., Shao G., Lam H.-M. (2008). Salt tolerance in soybean. J. Integr. Plant Biol..

[22] Qi X., Li M.-W., Xie M., Liu X., Ni M., Shao G., Song C., Kay-Yuen Yim A., Tao Y., Wong F.-L. (2014). Identification of a novel salt tolerance gene in wild soybean by whole-genome sequencing. Nat. Commun..

[23] Guan R., Qu Y., Guo Y., Yu L., Liu Y., Jiang J., Chen J., Ren Y., Liu G., Tian L. (2014). Salinity tolerance in soybean is modulated by natural variation in GmSALT3. Plant J..

[24] Yu Y., Wang N., Hu R., Xiang F. (2016). Genome-wide identification of soybean WRKY transcription factors in response to salt stress. Springerplus.

[25] Schmutz J., Cannon S.B., Schlueter J., Ma J., Mitros T., Nelson W., Hyten D.L., Song Q., Thelen J.J., Cheng J. (2010). Genome sequence of the palaeopolyploid soybean. Nature.

[26] Lam H.-M., Xu X., Liu X., Chen W., Yang G., Wong F.-L., Li M.-W., He W., Qin N., Wang B. (2010). Resequencing of 31 wild and cultivated soybean genomes identifies patterns of genetic diversity and selection. Nat. Genet..

[27] Kim M.Y., Lee S., van K., Kim T.-H., Jeong S.-C., Choi I.-Y., Kim D.-S., Lee Y.-S., Park D., Ma J. (2010). Whole-genome sequencing and intensive analysis of the undomesticated soybean (*Glycine soja* Sieb. and Zucc.) genome. Proc. Natl. Acad. Sci. USA.

[28] Belamkar V., Weeks N.T., Bharti A.K., Farmer A.D., Graham M.A., Cannon S.B. (2014). Comprehensive characterization and RNA-Seq profiling of the HD-Zip transcription factor family in soybean (*Glycine max*) during dehydration and salt stress. BMC Genom..

[29] Song H., Wang P., Hou L., Zhao S., Zhao C., Xia H., Li P., Zhang Y., Bian X., Wang X. (2016). Global analysis of WRKY genes and their response to dehydration and salt stress in soybean. Front. Plant Sci..

[30] Zhang D., Wan Q., He X., Ning L., Huang Y., Xu Z., Liu J., Shao H. (2016). Genome-wide characterization of the ankyrin repeats gene family under salt stress in soybean. Sci. Total Environ..

[31] Finn R.D., Clements J., Arndt W., Miller B.L., Wheeler T.J., Schreiber F., Bateman A., Eddy S.R. (2015). HMMER web server: 2015 Update. Nucleic Acids Res..

[32] Goodstein D.M., Shu S., Howson R., Neupane R., Hayes R.D., Fazo J., Mitros T., Dirks W., Hellsten U., Putnam N. (2012). Phytozome: A comparative platform for green plant genomics. Nucleic Acids Res..

[33] Finn R.D., Bateman A., Clements J., Coggill P., Eberhardt R.Y., Eddy S.R., Heger A., Hetherington K., Holm L., Mistry J. (2014). Pfam: The protein families database. Nucleic Acids Res..

[34] Hunter S., Apweiler R., Attwood T.K., Bairoch A., Bateman A., Binns D., Bork P., Das U., Daugherty L., Duquenne L. (2009). InterPro: The integrative protein signature database. Nucleic Acids Res..

[35] Filichkin S., Priest H.D., Megraw M., Mockler T.C. (2015). Alternative splicing in plants: Directing traffic at the crossroads of adaptation and environmental stress. Curr. Opin. Plant Biol..

[36] Crooks G.E., Hon G., Chandonia J.-M., Brenner S.E. (2004). WebLogo: A sequence logo generator. Genome Res..

[37] Cannon S.B., Mitra A., Baumgarten A., Young N.D., May G. (2004). The roles of segmental and tandem gene duplication in the evolution of large gene families in *Arabidopsis thaliana*. BMC Plant Biol..

[38] Severin A.J., Woody J.L., Bolon Y.-T., Joseph B., Diers B.W., Farmer A.D., Muehlbauer G.J., Nelson R.T., Grant D., Specht J.E. (2010). RNA-Seq Atlas of *Glycine ma*x: A guide to the soybean transcriptome. BMC Plant Biol..

[39] Jain M., Nijhawan A., Arora R., Agarwal P., Ray S., Sharma P., Kapoor S., Tyagi A.K., Khurana J.P. (2007). F-box proteins in rice. Genome-wide analysis, classification, temporal and spatial gene expression during panicle and seed development, and regulation by light and abiotic stress. Plant Physiol..

[40] Bailey T.L., Williams N., Misleh C., Li W.W. (2006). MEME: Discovering and analyzing DNA and protein sequence motifs. Nucleic Acids Res..

[41] Tamura K., Stecher G., Peterson D., Filipski A., Kumar S. (2013). MEGA6: Molecular Evolutionary Genetics Analysis version 6.0. Mol. Biol. Evol..

[42] Koralewski T.E., Krutovsky K.V. (2011). Evolution of exon-intron structure and alternative splicing. PLoS ONE.

[43] Carmel L., Wolf Y.I., Rogozin I.B., Koonin E.V. (2007). Three distinct modes of intron dynamics in the evolution of eukaryotes. Genome Res..

[44] Libault M., Farmer A., Joshi T., Takahashi K., Langley R.J., Franklin L.D., He J., Xu D., May G., Stacey G. (2010). An integrated transcriptome atlas of the crop model *Glycine max*, and its use in comparative analyses in plants. Plant J..

[45] Grant D., Nelson R.T., Cannon S.B., Shoemaker R.C. (2010). SoyBase, the USDA-ARS soybean genetics and genomics database. Nucleic Acids Res..

[46] Kuroda H., Yanagawa Y., Takahashi N., Horii Y., Matsui M. (2012). A comprehensive analysis of interaction and localization of *Arabidopsis* SKP1-like (ASK) and F-box (FBX) proteins. PLoS ONE.

[47] Lescot M., Déhais P., Thijs G., Marchal K., Moreau Y., van de Peer Y., Rouzé P., Rombauts S. (2002). PlantCARE, a database of plant cis-acting regulatory elements and a portal to tools for in silico analysis of promoter sequences. Nucleic Acids Res..

[48] Letunic I., Doerks T., Bork P. (2015). SMART: Recent updates, new developments and status in 2015. Nucleic Acids Res..

[49] Larkin M.A., Blackshields G., Brown N.P., Chenna R., McGettigan P.A., McWilliam H., Valentin F., Wallace I.M., Wilm A., Lopez R. (2007). Clustal W and Clustal X version 2.0. Bioinformatics.

[50] Gasteiger E., Gattiker A., Hoogland C., Ivanyi I., Appel R.D., Bairoch A. (2003). ExPASy: The proteomics server for in-depth protein knowledge and analysis. Nucleic Acids Res..

[51] Briesemeister S., Rahnenfuhrer J., Kohlbacher O. (2010). YLoc—an interpretable web server for predicting subcellular localization. Nucleic Acids Res..

[52] Liu R.H., Meng J.L. (2003). MapDraw: A microsoft excel macro for drawing genetic linkage maps based on given genetic linkage data. Hereditas.

[53] Hou X.-J., Li S.-B., Liu S.-R., Hu C.-G., Zhang J.-Z. (2014). Genome-wide classification and evolutionary and expression analyses of citrus MYB transcription factor families in sweet orange. PLoS ONE.

[54] Gu Z., Cavalcanti A., Chen F.-C., Bouman P., Li W.-H. (2002). Extent of gene duplication in the genomes of Drosophila, nematode, and yeast. Mol. Biol. Evol..

[55] Tang H., Bowers J.E., Wang X., Ming R., Alam M., Paterson A.H. (2008). Synteny and collinearity in plant genomes. Science.

[56] Librado P., Rozas J. (2009). DnaSP v5: A software for comprehensive analysis of DNA polymorphism data. Bioinformatics.

[57] Lynch M., Conery J.S. (2000). The evolutionary fate and consequences of duplicate genes. Science.

[58] Meng X., Wang C., Rahman S.U., Wang Y., Wang A., Tao S. (2015). Genome-wide identification and evolution of HECT genes in soybean. Int. J. Mol. Sci..

[59] CRAN—Package Pheatmap. https://cran.r-project.org/web/packages/pheatmap/.

[60] Livak K.J., Schmittgen T.D. (2001). Analysis of relative gene expression data using real-time quantitative PCR and the 2(-Delta Delta C(T)) Method. Methods.

[61] Yim A.K.-Y., Wong J.W.-H., Ku Y.-S., Qin H., Chan T.-F., Lam H.-M. (2015). Using RNA-Seq data to evaluate reference genes suitable for gene expression studies in soybean. PLoS ONE.

