# Supplementary material for: Genome-Wide Analyses of the Soybean F-Box Gene Family in Response to Salt Stress
Source: Int J Mol Sci. 2017 Apr 12;18(4):818. doi: 10.3390/ijms18040818 (PMC5412402; doi:10.3390/ijms18040818)

```
GmFBX1       --------------FSSLPYDVLAKIAASFD--DPNLR-------AASLVCRAWCEA--------------------------------------------------------------------  34
GmFBX12.1    ------PSPPGAGDFSALPYDVLAKIAASFD--DPNLR-------AASLVCRAWCEAL-------------------------------------------------------------------  43
GmFBX84      -----------------------MQILSLVD--DQTVI-------TASGVCRGWRDA--------------------------------------------------------------------  25
GmFBX122.1   --------------WKDIPVELLMQILSLVD--DQTVI-------IASGVCRGWRDA--------------------------------------------------------------------  34
GmFBX287.1   ---------------KDIPVELLMQILSLVD--DQTVM-------IASEVCRGWREAI-------------------------------------------------------------------  34
GmFBX330.1   ---------------KDIPVELLMQILSLVD--DQTVI-------IASEVCRGWREAI-------------------------------------------------------------------  34
GmFBX14      --PRLDDSLSESSKLESLPMDLLVKILCHLH--HDQLR-------AVFHVSQRIRKAVIMARQFHFNN---------------------------------------------------------  57
GmFBX368     --PRLDDSLSES-KLESLPMDLLVKILCHLH--HDQLR-------AVFHVSQKIRKAVIIARQFHFNYTT-------------------------------------------------------  58
GmFBX155     ------TFDSERSRLEALPLDVLIRVLCGVD--HEDLE-------QLVRVSKTVREAAEIARR--------------------------------------------------------------  48
GmFBX168     --------DSERSRLEALPLDVLIRVLCGVD--HEDLK-------QLVRVSKTVREAAEVARR--------------------------------------------------------------  46
GmFBX308     ----------KRSLLDALPQDVLVQVLCGVD--HEDLK-------QLFHVSKTIREATTVVKKLHFDISTPKKKT--------------------------------------------------  56
GmFBX5       ------------------------------N--VVDLA-------VASLVCKMWNKACH-DPSLWRKID--------------------------------------------------------  29
GmFBX16      --------------------DIIITILMSLN--VVDLA-------VASLVCKMWNKACR-DPSLWRKID--------------------------------------------------------  39
GmFBX352     -------------------TEILITIFMSVN--IVDLA-------VASLVCKMWNTACR-DPSLWGKID--------------------------------------------------------  40
GmFBX237     --------------------EILVTILMFLN--VGDIA-------AASMVNKIWNKACH-DPSLWHTLD--------------------------------------------------------  39
GmFBX508     -------------TILSLDPDIVTTIFAFLD--MFDLV-------RCSLVCKLWNAIVE-SRSLRE-----------------------------------------------------------  43
GmFBX6       ---------------SQIPPELFHHILKFLS--SEDLV-------SCSLVCRFLNYAAS-DEALWRRLY--------------------------------------------------------  44
GmFBX271     ---------------SQIPPELFNHILKFLS--SEDLV-------SCSLVCRFLNYAAS-DEALWRRLY--------------------------------------------------------  44
GmFBX263     -----------------LPQDILVLIFSFLD--MKSLV-------SVGLVCRSWNIAAN-DNHLW------------------------------------------------------------  38
GmFBX497     ----------------QLPQDILAHIFSFLE--MKSLV-------SMGLVCWSWNIAAN-DNHLWEKQ---------------------------------------------------------  42
GmFBX41      --------------------DILREILKRLD--GPSLG-------VAACVCRLWCSLTSNDDSLWEHLCF-------------------------------------------------------  41
GmFBX319     --------------------DILREILKRLD--GPSLG-------VAACVCRLWCSLTRNDDSLWEHLCF-------------------------------------------------------  41
GmFBX253.1   --------------------DILRLIFDNLP--IPDLA-------RASCVCRLWNSVA-------------------------------------------------------------------  29
GmFBX504     --------------------DILRLIFENLP--IIDLA-------RASCVCRLWCSVA-------------------------------------------------------------------  29
GmFBX83      ----------------MLPLEICMKIFCLLD--YQHLA-------VAQQVCRKWKLVAS-DNTLWSNL---------------------------------------------------------  42
GmFBX124     ---------------ERLPLEICMKIFYLLD--YQHLA-------VAQQVCRKWKLVAS-ENALWSDL---------------------------------------------------------  43
GmFBX27      ----------------GLPARLLWEVMRRLP--PPGLL-------SAAKVCKGWRDTAK-RMW--------------------------------------------------------------  37
GmFBX240     -----------------LPASLMWEVMRRLP--PPGLL-------SAAKVCKGWRDTTK-R----------------------------------------------------------------  34
GmFBX53      ---------------SGLPGNLLWEVLRRLP--PAGLL-------SAAKVSRGWREMTR-S----------------------------------------------------------------  36
GmFBX475     ---------------SGLPGNLLWEVLRRLP--PAGLL-------TAAMVSRGWREMTR-S----------------------------------------------------------------  36
GmFBX85.1    ---------------RSLPDELLFEVFARMT--PYDLG-------RASCVCRKWKYTIR-NPVFWRNACL-------------------------------------------------------  45
GmFBX121     ---------------RSLPDELLFEVFARMT--PYDLG-------KASCVCRKWKYTIR-NPVFWRNACL-------------------------------------------------------  45
GmFBX69      -------------------DNLLFEVLKHVD--ARTLA-------MSGCVNKQWHKTAQ-DERLWELI---------------------------------------------------------  39
GmFBX111     -------------------DNLLFEVLKHVD--ARTLA-------MAGCVNKQWHKTAQ-DERLWELI---------------------------------------------------------  39
GmFBX325     -------------------DNVLSEVLRHVD--ARSLA-------MAGCVSKQWQKMAR-DERLWELIC--------------------------------------------------------  40
GmFBX405     ------------------DDNVLFEVLKHVD--ARSLA-------MAGCVSKQWQKAAR-DERLWELI---------------------------------------------------------  40
GmFBX32      ------------------PWEALILVATYLD--PKTLA-------MASCVSKSWFSSMS-SDIIWKPILATHFPSLSTLPSSAPTVAYRRL----------------------------------  63
GmFBX327     -------------------DDLLHMVFSFLD--HPNLC-------KAARICKQWRGASA-HEDFWKSLN--------------------------------------------------------  40
GmFBX403.1   -------------------DDLLHMVFSFLD--HPNLC-------KAARVCKQWRGASA-HEDFWKSLN--------------------------------------------------------  40
GmFBX113     -------------------DDLLHMVFSFLD--HPNLC-------RAARVCKQWRTASA-HEDFWKSLN--------------------------------------------------------  40
GmFBX71.1    -------------------DDLLHMVFSFLD--HPNLC-------KAARVCKQWWTASA-HEVFWKSLN--------------------------------------------------------  40
GmFBX193.1   -------------------EEVLKAVFPFLD--SVDLA-------SCMGVCTQWKDIAS-DDFFWKCL---------------------------------------------------------  39
GmFBX445     -------------------EEVLKAVFPFLD--GVDLA-------SCMAVDKQWKDIAS-DDFLWKCL---------------------------------------------------------  39
GmFBX2       --------------------DILEAIFSHVP--LIHLV-------PASHVSNSWKRAVSSSL---------------------------------------------------------------  33
GmFBX11      --------------------DILEAIFSHVP--LIHLV-------PASHVSNSWKRAVSSSL---------------------------------------------------------------  33
GmFBX200     --------------------DILEAIFSHVP--LIHLV-------PASHVSNSWKRAVSTSLR--------------------------------------------------------------  34
GmFBX413     --------------------DILEAIFSHVP--LIHLV-------SASHVSHEWKRAIS------------------------------------------------------------------  30
GmFBX4       ---------------------ILIEILSHVP--AKDLL-------SLKRVCKEWHHVIS------------------------------------------------------------------  29
GmFBX198     -----------------LNSDILIEILSHVP--AKDLL-------SLKLVSKEWHRLISS-----------------------------------------------------------------  34
GmFBX63      -------------------DDILHEILLRIP--PPTIS-------KLIIVSKIWLRVICSPSFRQCYVRQWGQSFRLLGF---------------------------------------------  52
GmFBX482     -------------------DDILHEILLRIP--PPTIS-------KLIIVSKIWLRVICSPSFRQCYLRQWGQSFRLLGF---------------------------------------------  52
GmFBX141     -------------SINNLSDDVMGEIFIRLP--FRSTV-------KCKCVCKRWLGLISSPSFPTE-----------------------------------------------------------  44
GmFBX188     -------------NINNLLDDVLGEIFIRLP--FRSTN-------TCKCVCKRWLGLISS-----------------------------------------------------------------  38
GmFBX215     -------------TINNLFDDSLTEIFCKLP--CKSLF-------TCKSVSKRWLTLISNPNFHSLF----------------------------------------------------------  45
GmFBX21      ----------------RLAEDSLRQIFCRLP--LREIM-------ICRSVSKFFLQLLSTPS---------------------------------------------------------------  37
             1.......10........20........30........40........50........60........70........80........90.......100.......110.......120.....
```

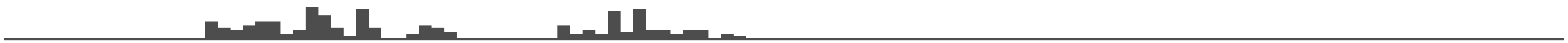

```
GmFBX82      -------SESPNNLIPYLPNDVALNCLARIP--RSHHP-------TLSLVSKPIRSLLYSPLLFTTRSLLQCTQPLLYLTLRSRDSSLQWFTLHRTNPNPLLAPLP-------------------   90
GmFBX125     --------------IPSLPDDVALNCLGRIP--RSQHP-------TLSLVSKPIRTLLSSPILF-------------------------------------------------------------   41
GmFBX52.1    --------------IERLPDAVAIRCLARVP--FYFHP-------VLELVSRSWQAAIRSPEL--------------------------------------------------------------   40
GmFBX147.1   --------------ICGLPDDISLMCLARIP--RKYHS-------VMKCVSKRWRNLICSEEW--------------------------------------------------------------   40
GmFBX148     --------------ICGLPDDISLMCLARVP--RKYHS-------VLKCVSKRWRDLICS-----------------------------------------------------------------   37
GmFBX146     --------------ICGLPDDLSLMCLARVP--RKYHS-------VLKCVSKRWRDLICS-----------------------------------------------------------------   37
GmFBX144     -------------LISGLPEDVARDCLIRVS--YQQFP-------TVASVCKLWKSEIHAPEFRRQRRSTKHAQK--------------------------------------------------   53
GmFBX186     --------------ISGLPEDVARDCLIRVS--YQQFP-------TVASVCKLWKSEIHAPEFHR------------------------------------------------------------   42
GmFBX318     --------------ISGLPEDVARDCLIRIP--YEQFP-------AVASVCKGWNTEIHSPDFHRR-----------------------------------------------------------   43
GmFBX335     --------------ISGLPEDVARDCLIRVP--YDQFP-------AVASVCKGWSAEIHSPDFHR------------------------------------------------------------   42
GmFBX277     --------------LPGLPDDVAEYCLALVP--RSNFP-------AMGGVCKIWRSFIQSK----------------------------------------------------------------   38
GmFBX411     --------------LPGLPDDVAEYCLALVP--RSNFP-------AMGVVCKGWRSFIQSKE---------------------------------------------------------------   39
GmFBX174.1   --------------LPGLPDDVSKHCLALVP--RSNFP-------AMGGVCKRWRGFIRSKE---------------------------------------------------------------   39
GmFBX284     -------------LLPGLIDDVALNCLAWVS--GSDYA-------ALSCINKRFNKLINSGYLYGLRKQLGAVEHLVYMVCDPRGWVAFDPKINRWMSLPKIPCDECF-----------------   86
GmFBX316.1   ------------SLIPGLIDDVALNCLAWVS--GSDYA-------VLSCINKRFNKLINSGYLYGLRKQLGAVEHLVYMVCDPRGWVAFDPKINRWISLPKIPCDEC------------------   86
GmFBX54      --------------IPGLPNDVAASILSKVP--YSHHG-------RLKATCKSWKLLLSSKS---------------------------------------------------------------   39
GmFBX476     --------------IPGLPNDVAASILSMVP--YSHHG-------RLKATCKSWKLLLS------------------------------------------------------------------   36
GmFBX86      --------------LPGLPDDLAIACLIRVP--RVEHR-------KLRLVCKRWYRLL-------------------------------------------------------------------   35
GmFBX453     --------------LPGLPDDLAIACLIRVP--RVEHR-------KLRLVCKRWYRLLV------------------------------------------------------------------   36
GmFBX99.1    --------------LPGLPDDLAIACLIRVP--RVEHS-------KLRLVCKRWYRLLSGNF---------------------------------------------------------------   39
GmFBX177     --------------LPGLPDDLAIACLIRVP--RVEHG-------KLRLVCKRWYHLLSGNF---------------------------------------------------------------   39
GmFBX212     -------------LLSGLPDDLAIACLIRVP--RIEHR-------KLHLVCKRWHRLLSEDFF--------------------------------------------------------------   41
GmFBX350.1   --------------LPGLPDDLAVTCLIRVP--RIEHR-------KLHLVCKRWRRLLSEDFF--------------------------------------------------------------   40
GmFBX303     --------------IPGLPDDVALNCLLRLP--VQSHS-------SCRAVCKRWHMLLGNK----------------------------------------------------------------   38
GmFBX344     --------------IPGLPDDVALNCLLRLP--VQSHS-------SCRAVCKRWHMLLGNK----------------------------------------------------------------   38
GmFBX488     -------------TISSLPDDIVLDCLSRVP--TSSLP-------ALSLVCRRWSRLLSSPD---------------------------------------------------------------   40
GmFBX75      --------------IPNLPDELSLQIIARLP--RICYF-------NVRLVSKRWKSTIMSSELY-------------------------------------------------------------   41
GmFBX120.1   --------------IPNLPDELSLQIIARLP--RICYF-------NVRLVSKKWKSTIMSSELY-------------------------------------------------------------   41
GmFBX290     --------------IPNLPDELSLQIIARLP--RICYY-------HVRLVSKRWKATITSSELY-------------------------------------------------------------   41
GmFBX332     --------------IPNIPDELSLQIIARLP--RICYY-------HVRLVSRRWKTTITSLELY-------------------------------------------------------------   41
GmFBX353     --------------IPNLPDDLSLQIIARLP--RICYY-------HVRLVSRRWKATIKFNLIYDRLCI--------------------------------------------------------   46
GmFBX94.1    --------------IPSLPDEISIQILARVP--RIYYL-------NLKLVCRAWKETLVSSELF-------------------------------------------------------------   41
GmFBX169.1   --------------IPSLPDEISIQILARVP--RIYYL-------NLKLVCRAWKET--------------------------------------------------------------------   34
GmFBX100     ------------DLIPKLPSELGLECLTRLP--HSAHR-------VALRVCSQWHCLLQSDAFYS------------------------------------------------------------   44
GmFBX116     -------------LIPGLPYEIAELCLLHVP--YPYQA-------LSRSVSSTWNRAITHPSFIYSKKTLSHPHLFVLAFHSQTGKIQWQALDPSSGRWF-------------------------   78
GmFBX66.1    -----------------LPDDILEMCLVRLP--LTSLM-------NARLVCKKWRSLTTTPRF--------------------------------------------------------------   37
GmFBX109.1   -----------------LPDDILEMCLVRLP--LTSLM-------NARLVCKKWRSLTTTPRF--------------------------------------------------------------   37
GmFBX70      --------------WSKLPPDVVEHILLLLP--LKTLL-------NLRPTCKAFTSLLFSPSFVSK-----------------------------------------------------------   43
GmFBX112     --------------WSKLPPDVVEHILLLLP--LKTLL-------NLRPTCKAFTCLLFSPSFVSK-----------------------------------------------------------   43
GmFBX326     --------------WSKLPPEILEYILSFLP--LKTFL-------NLRSTCKGFWSLIFSPPFISKH----------------------------------------------------------   44
GmFBX10      -----------------LPEDLITEILMMLP--VRSIL-------RFKCMCKSWFSLISDPE---------------------------------------------------------------   36
GmFBX379     -----------------LPEDLITEILMMLP--VRSIL-------RFKCMCKLWFSLISDPE---------------------------------------------------------------   36
GmFBX377     -----------------LPEDLITEILMMLP--VRSIL-------RFKYMCKSWFSLISHPE---------------------------------------------------------------   36
GmFBX380     -----------------LPEDLITEILMMLP--VRSIL-------RFKCMCKSWFFLISHPE---------------------------------------------------------------   36
GmFBX378     --------------------DLITEILMMLP--VRSIL-------RFKCMCKLWFSLISHPEFARSHFALAATPT--------------------------------------------------   46
GmFBX19      -----------------LPQELIIEILLRLP--VKSLV-------RFKCVCRLWLSLISDPS---------------------------------------------------------------   36
GmFBX371     -----------------LPQELIIEILLRLP--VKSLV-------RFKCVCKLWLSLISDPH---------------------------------------------------------------   36
GmFBX123     --------------IAILPWELIIEILLRLP--VKSLV-------RFKCVCKSWLCLLSDPH---------------------------------------------------------------   39
GmFBX292     -----------------LPEELIIEILLRLP--VKSLL-------RFKRVCNSWLSLIS------------------------------------------------------------------   33
GmFBX448.1   ----------------TLPQELIREILLRLP--VKSLL-------RFKCVCKSFLSLISDPQF--------------------------------------------------------------   38
GmFBX157     -----------------LPQELIIQILLRLP--VKSLL-------RFKCVSKSWLSLITDPH---------------------------------------------------------------   36
GmFBX389     ---------------VFLPQELIIQILLRLP--VKSLI-------RFKCIS--------------------------------------------------------------------------   27
GmFBX171.1   -----------------LPQDLITEILLRLP--VKSLV-------RFKSVCKSWLFLISDPR---------------------------------------------------------------   36
GmFBX385     -----------------LPQDLVIQILLRLP--VKSLV-------RFKSVCKSWLSFISDSHLWLS-----------------------------------------------------------   40
GmFBX382     ----------------DLPQELIHEILLRLP--VKSLI-------RFKTVCKSWLSHISDPH---------------------------------------------------------------   37
             1.......10........20........30........40........50........60........70........80........90.......100.......110.......120.....
```

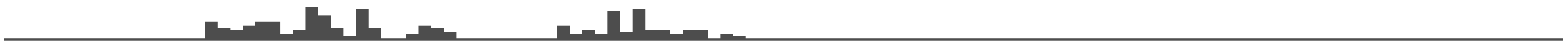

```
GmFBX383    -----------------LPQEMVNQILLRLP--VKSLL-------QFKTVCKSWLSHISDPH---------------------------------------------------------------   36
GmFBX198.1  -----------------LPLELIREVLLRLP--VRSVL-------RFRCVCKSWLSLISDPQF--------------------------------------------------------------   37
GmFBX245    -----------------LPLELIREILLRLP--VRSVL-------RFKCVCKSWLSLISDPQ---------------------------------------------------------------   36
GmFBX449    -----------------LPLELIIEILMRLP--VRSVL-------GFKCVCKSWFSLISDPQ---------------------------------------------------------------   36
GmFBX152    ----------------SLPLELIEEILLRLP--VRSIL-------RFKCVCKSWFSLISEP----------------------------------------------------------------   36
GmFBX45.1   ------------------PGELIGAILLWLP--VRSVL-------RFKCVCKSWLSVISDPH---------------------------------------------------------------   35
GmFBX447.1  -----------------LPLDLIELILLKLP--VKSVT-------RFKCVCKSWLSLISDPQF--------------------------------------------------------------   37
GmFBX194    -----------------LPMDLMREILLRLP--VRSVS-------RFKCVCKSWLSIISDPQ---------------------------------------------------------------   36
GmFBX446.1  -----------------LPMELMREILLRLP--VRSVS-------RCKCVCKSWNFIISNP----------------------------------------------------------------   35
GmFBX246    -----------------LPDELIVEILLRLP--VRTLL-------RFKCVRKSWLFLISDPQF--------------------------------------------------------------   37
GmFBX397    -----------------LPDDQIVEILLRLP--VRTLL-------RFKCVRKSWLFLISDPQFTKSHFDLAA-----------------------------------------------------   46
GmFBX489    -----------------LPFDLIVEILLRLS--VRSLL-------RFKCVSKSWCALISDPE---------------------------------------------------------------   36
GmFBX108    ---------------PMLPDDLIVNILSRLR--VRSLM-------RSKCVCKSWLSLISDPQFVK------------------------------------------------------------   41
GmFBX193    ------------------SHDLIVEILLRLP--IKSLL-------RFKCVCKSWLSFISDPHFVK------------------------------------------------------------   38
GmFBX130    -----------------LPDELMEEILLRLP--VRCLV-------RFKCVCKSWLSLISDPQ---------------------------------------------------------------   36
GmFBX131    -----------------IPDDMMEEILLRLP--VKCLL-------RFKYVCKSWLSLISDPH---------------------------------------------------------------   36
GmFBX154    -------------------DDLTIEILLRLP--VRCLL-------RFKCVCKSWFSLISNPE---------------------------------------------------------------   34
GmFBX192    -----------------LPRDLIREILLRLP--VKSVL-------KCKRVCKTWLSLISDPKF--------------------------------------------------------------   37
GmFBX195    -----------------LPPELIREILLSLP--VNSLL-------QCKRVCKAWLSLISDPQFF-------------------------------------------------------------   38
GmFBX132    -----------------LPEELIQVILLRLP--LRNLL-------HLKRVCKSWLSLISDPQ---------------------------------------------------------------   36
GmFBX31     -------------------EELISNILHRVP--VRSLL-------QFKCVCKSWNSLISDPL---------------------------------------------------------------   34
GmFBX260    -------------PLWLLPEELISEILFRVP--VRSLL-------QFRCVCKSWKTLISHP----------------------------------------------------------------   39
GmFBX261    ---------------AVLPYDLIWEILLRVP--VRSLI-------LFKCVCKSWKTLISDPQ---------------------------------------------------------------   38
GmFBX300.1  ---------------PFLPDELVVEILSRLP--VKSLL-------QFRCVCKSWMSLISDPYFMKK-----------------------------------------------------------   42
GmFBX347    ---------------PFLPDELVVEILSRLP--VKSLL-------QFRCVCKSWMSLIYDPYFMKK-----------------------------------------------------------   42
GmFBX348    -----------------LPIELIQEILQRLP--VKFLL-------QLRCVCKSWKSLISHPQ---------------------------------------------------------------   36
GmFBX417    ---------EKKPWSSLLCNEIIEEILSHLP--VKPLI-------QFKCVRKEWNSLMSEPYFIKLHLCKSA-----------------------------------------------------   54
GmFBX429    ---------------SLLCNKIIEEILSRFP--VKPLI-------QFKCVCKEWNSLMSEPY---------------------------------------------------------------   38
GmFBX331    -------------WLSLLCNEIIEEILSRLP--VKPLI-------QFMCVCKEWNSLMSEP----------------------------------------------------------------   39
GmFBX427    ---------------SLLCNEIIEEILSRLP--VKPLI-------PFKCVCKGWNSLMSEPY---------------------------------------------------------------   38
GmFBX422    -----------------LCNEIIKEILSRLP--VKPLI-------QFKCVCKEWNSLISEPYF--------------------------------------------------------------   37
GmFBX425    ---------------SLLCNEIIKEILSRLP--VKPLI-------QFKCMCKEWNSLISEPYF--------------------------------------------------------------   39
GmFBX439    ---------------SLLCNEIIKEILSRLP--VKPLI-------KFKCVCKEWNSLISEPYF--------------------------------------------------------------   39
GmFBX437    ------------------------EILSRLP--MKPLI-------QFKCVCKEWNSLISEPYF--------------------------------------------------------------   30
GmFBX414    -----------------LCDELIKEILSRLP--VKPLI-------QFKCVCKGWNSLMSDPYF--------------------------------------------------------------   37
GmFBX423    -----------------LCDELIKEILSRLP--VKPLI-------QFKCVCKGWNSLMSDPYF--------------------------------------------------------------   37
GmFBX507    -----------------LFDELIKEILSRLP--VKPLI-------QFKCVCKGWNSLMSDPYF--------------------------------------------------------------   37
GmFBX418    -------------------DELIKEILSCLP--VKTLI-------QFKCVYKGWNSLMSDPY---------------------------------------------------------------   34
GmFBX436    -------------------DELIKEILSRLP--VKTLI-------QFKCVYKGWNSLMSDPY---------------------------------------------------------------   34
GmFBX424    -----------------LCDELIKEILSRLP--VKPLI-------QFKCVYKGWNSLMSDPY---------------------------------------------------------------   36
GmFBX416    -----------------LCDELIEEILSRLP--VKPLI-------QFKCVCKGWNSLMSDPY---------------------------------------------------------------   36
GmFBX420    -------------------DELIEEILSRLP--VKPLI-------QFKCVCKGWNSLMSDPY---------------------------------------------------------------   34
GmFBX438    -----------------LCDELIEEILSRLP--VKPLI-------QFKCVCKGWNSLMSDPY---------------------------------------------------------------   36
GmFBX435    -----------------LCDELIEKILSRLP--VKPLI-------QFKCVCKGWNSLMSDPY---------------------------------------------------------------   36
GmFBX506    -----------------LCDELIEQILSRLP--VKPLI-------QFKCVCKGWNSLMSDPY---------------------------------------------------------------   36
GmFBX421    -----------------LCDELFEEILSRLP--VKPLI-------QFKCVCKGWNSLMSDPY---------------------------------------------------------------   36
GmFBX430    -----------------LCDELIEEILSRIL--VKPLI-------QFKCVCKGWNSLMSDPY---------------------------------------------------------------   36
GmFBX441    -----------------LCEELIEEILSRLP--VKPLI-------QFKCVCKGWNSLMSDPY---------------------------------------------------------------   36
GmFBX419    -----------------LCDKLIEEILSRLP--VKPFI-------QFKCVCKGWNSLMSDPY---------------------------------------------------------------   36
GmFBX428    -----------------LCDELIEEILSRLP--VKPFI-------QFKCVCKGWNSLMSDPYF--------------------------------------------------------------   37
GmFBX415    -----------------------MEILSRLP--VKPLI-------QFKCVYKGWNSLMLDPYFIKLHLNKSA-----------------------------------------------------   40
GmFBX440    -----------------------MEILSRLP--VKPLI-------QFKYVCKGWNSLMSDPY---------------------------------------------------------------   30
GmFBX426    -----------------LCDELFEEILSRLP--VKPLM-------QFKCVCKGWNSLISDPYF--------------------------------------------------------------   37
GmFBX451    -------------MNTYIPRELTEKILIKLP--VKSLV-------SFKCVRKEWNNLISDPEFAERHFKYGQRAETLMITTPDVNHFKSI-----------------------------------   68
GmFBX505    -------------MNTYIPRELTEKILIKLP--VKSLV-------SFKCVRKEWNNLISDPEFAERHFKYGQRAETLMITTPDVNHFKSI-----------------------------------   68
            1.......10........20........30........40........50........60........70........80........90.......100.......110.......120.....
```

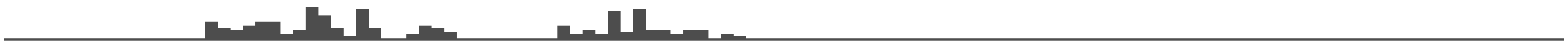

```
GmFBX213     ------------------PDEVVIQILARLP--VKSLF-------RFKTVCKLWYRLSLD-----------------------------------------------------------------   33
GmFBX339     -----------------LPYDVIINILKRLP--VKSLI-------RFKCVSKDWFNL--------------------------------------------------------------------   31
GmFBX364     ----------------HLPQELVSNILSRLP--AKDLV-------KCKRVCKSWFDLIT------------------------------------------------------------------   34
GmFBX468     ----------------HLPQELVSNILSRLP--AIDLV-------KCKSVCKSWFDLITDSHFV-------------------------------------------------------------   39
GmFBX365     ------------MSMEHLPGELVSNVLSRLP--SKVLL-------LCKCVCKSWFDLITDPHFV-------------------------------------------------------------   43
GmFBX467     ------------MSMEHLPRELVSNVLSRLP--AKVLL-------LCKCVCNSWFDLITDPHFV-------------------------------------------------------------   43
GmFBX13      -----------------LPEDLIVEILSWVE--VKNLM-------RFRCVSKSWNSLIF------------------------------------------------------------------   33
GmFBX101     -----------------LSEDLIVEILTWVP--VKSLM-------RFRCVSKSWNSLIFH-----------------------------------------------------------------   34
GmFBX89      ---------------SLLPEELIVEILSWVP--VKALM-------QFRCISKTWNSLILHPT---------------------------------------------------------------   38
GmFBX91      ---------------SLLPEELIVEILSWVP--VKALM-------QFRCVSKTWNSLILHPT---------------------------------------------------------------   38
GmFBX209     ---------------SLLPEELIAEILSWVP--VKALM-------QFRCVSKTWNSLILHPT---------------------------------------------------------------   38
GmFBX179     -----------------LPEELIVEILSWVP--VKPLM-------RFKCVSKTWNSLIFH-----------------------------------------------------------------   34
GmFBX484     ---------------SILPQDQIVEILSWHP--VKVLM-------RFRCVSKTWNSLILNPT---------------------------------------------------------------   38
GmFBX191     -----------------LPRELIVEILSWLP--VKALM-------RFRYVSETWNSLIFDPT---------------------------------------------------------------   36
GmFBX129     -------------------DEVVVEILSWVP--VKALM-------RFRCVSKSWNSLILDPTF--------------------------------------------------------------   35
GmFBX88      ------------------PIELIVEILSWLP--VKPLI-------RFRCVSKTWKSLISH-----------------------------------------------------------------   33
GmFBX90      ------------------PIELIVEILSWLP--VKPLI-------RFRCVSKTWKSLISH-----------------------------------------------------------------   33
GmFBX208     ------------------PIELIVEILSWLP--VKPLI-------RFRCVSKTWKSL--------------------------------------------------------------------   30
GmFBX199     -----------------LPQELIVEILSWLP--VKPLM-------RFRCVSKAWYSLIFHPS---------------------------------------------------------------   36
GmFBX394     -------------------EDLSVEILSWLP--VKVLI-------RFKCVSKTWNSLIFHPML--------------------------------------------------------------   35
GmFBX293     ------------------------KILSWLP--VKALL-------RFRCVCKSWKSLMLDLSFV-------------------------------------------------------------   31
GmFBX390     -----------------LPGDLIVEILSWLP--VDALL-------RFRCVCKSWKSLIFDPSF--------------------------------------------------------------   37
GmFBX205     ---------------SYVPDDLIVEILSRLP--VKDLM-------RFRCVCKTWKSIIFDPSFVKK-----------------------------------------------------------   42
GmFBX455     ---------------AQLPQDLIEEILSWLP--VKSLM-------RFRCVSRTWNSL--------------------------------------------------------------------   33
GmFBX458     ---------------AQLPQDLIEEILSWLP--VKSLM-------RFRCVSRTWNSL--------------------------------------------------------------------   33
GmFBX460     ---------------AQLPQDLIEEILSWLP--VKSLM-------RFRCVSRTWNSL--------------------------------------------------------------------   33
GmFBX461     ---------------AQLPQDLIEEILSWLP--VKSLM-------RFRCVSRTWNSL--------------------------------------------------------------------   33
GmFBX462     ---------------AQLPQDLIEEILSWLP--VKSLM-------RFRCVSRTWNSL--------------------------------------------------------------------   33
GmFBX456     ---------------AQLPQDLIEEILAWLP--VKSLM-------RFRCVSRTWNSL--------------------------------------------------------------------   33
GmFBX457     ---------------AQLPQDLIEEILAWLP--VKSLM-------RFRCVSRTWNSL--------------------------------------------------------------------   33
GmFBX464     ---------------AQLPQDLIEEILSWLP--VKSLM-------RFRCVSSTWNSL--------------------------------------------------------------------   33
GmFBX459     ---------------AQLPQDLIEEILSWLP--VKSFM-------RFRCISRTWNSL--------------------------------------------------------------------   33
GmFBX463     -----------------LPRELLVEILSWLP--VKSLL-------RIRCVFKAWNSF--------------------------------------------------------------------   31
GmFBX153     ----------KFHVMEILFEKLMMEVLSWLP--VKSLV-------RFTCASKWFQSLISDSSFVKLHLQRSPKSEDFLLICSVDDTLNRFFILSCPAIPLVSDD---------------------   85
GmFBX490.1   ----------------ILPEELLVEILSWVP--VKDLL-------RFRCVAKWLRALISDPTFV-------------------------------------------------------------   39
GmFBX162.1   ---------------ANLPVEVVTEILSRLP--VKSVI-------RLRSTCKWWRSIIDSRHFV-------------------------------------------------------------   40
GmFBX381.1   --------------MANLPVEVVTEILSRLP--VKSVI-------RLRSTCKWWRSIIDSRH---------------------------------------------------------------   39
GmFBX211.1   ----------------HLPREVVTDILSRLP--AKSLL-------RFRSTSKSWKSLIDSQHF--------------------------------------------------------------   38
GmFBX349.1   ----------------HLPREVLTEILSRLP--VRSLL-------RFRSTSKSWKSLIDSQHLNW------------------------------------------------------------   40
GmFBX26.1    ---------------NLPDEIMLFKILPLLP--SKTLI-------RFRCVCKLWDCFIRDPSFLHLRKLTNNPTHHFLF----------------------------------------------   55
GmFBX294     ----------------------------WLP--EKTLL-------RLRCVCKFWKTLVFDPI---------------------------------------------------------------   25
GmFBX258     ------------------PDEILVEILHRLP--SKSIL-------RCSAVCKSWRSLISN-----------------------------------------------------------------   33
GmFBX499     ------------------PDEILVEIFRRLP--SKSIV-------RCSAVCKLWRSLVTN-----------------------------------------------------------------   33
GmFBX370     -------------------DEILGEILHRLP--AKTLL-------KCTTVCKSWKSLITHPSFISTI----------------------------------------------------------   39
GmFBX412     ---------------NHIPYDLTFFILSKLP--SKSLK-------KFGCVHKLWTLVFQNLYFMSMF----------------------------------------------------------   43
GmFBX43      -----------------LSHDELLEIFSRLP--AKAIY-------RFTSTSKSFSKLPKETYFASKQTQNSLLRDDTCFFIQPYITQSYNVHVEF------------------------------   69
GmFBX342     ------------------PDELLFKICSYLP--AKAIY-------RFKCTCKTVSNIVEETEFATKQTENS------------------------------------------------------   45
GmFBX79      ----------------LLPQELIQNIFLSLV--LPEII-------RLKLVNKSFSRIISDHAF--------------------------------------------------------------   38
GmFBX133     ----RNSTTKNAVGLCLLPQELIQNIFLSLV--LPEII-------RLKLLNKSFSRIISDNTFVRQYNSLSTSTTWLFVYKKR------------------------------------------   70
GmFBX486     --------------SVVLPLELITEIFLRLP--LKSLI-------RFKCVC--------------------------------------------------------------------------   28
GmFBX65      --------------LDVLPDDLLERILAYLP--IASIF-------RAGCVSKRWHEIVNSERFV-------------------------------------------------------------   41
GmFBX483     --------------LDVLPDDLLERILAYLP--IASIF-------RAGCVSKRWHEIVNSERFV-------------------------------------------------------------   41
GmFBX95      --------------WSKLPQRLLDRVIAFLP--PPAFF-------RARCVCKRWYAL--------------------------------------------------------------------   34
GmFBX170     --------------WSKLPQRLLDRVLAFLP--PPAFF-------RARCVCKRWYAL--------------------------------------------------------------------   34
GmFBX72      ------------FSLDDLNEDLFERILSWLP--TSTFF-------RLNSVCKRWKSVAASA----------------------------------------------------------------   40
             1.......10........20........30........40........50........60........70........80........90.......100.......110.......120.....
```

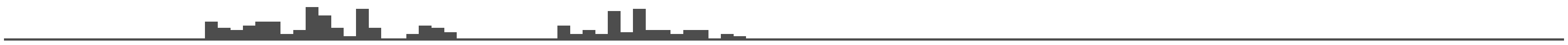

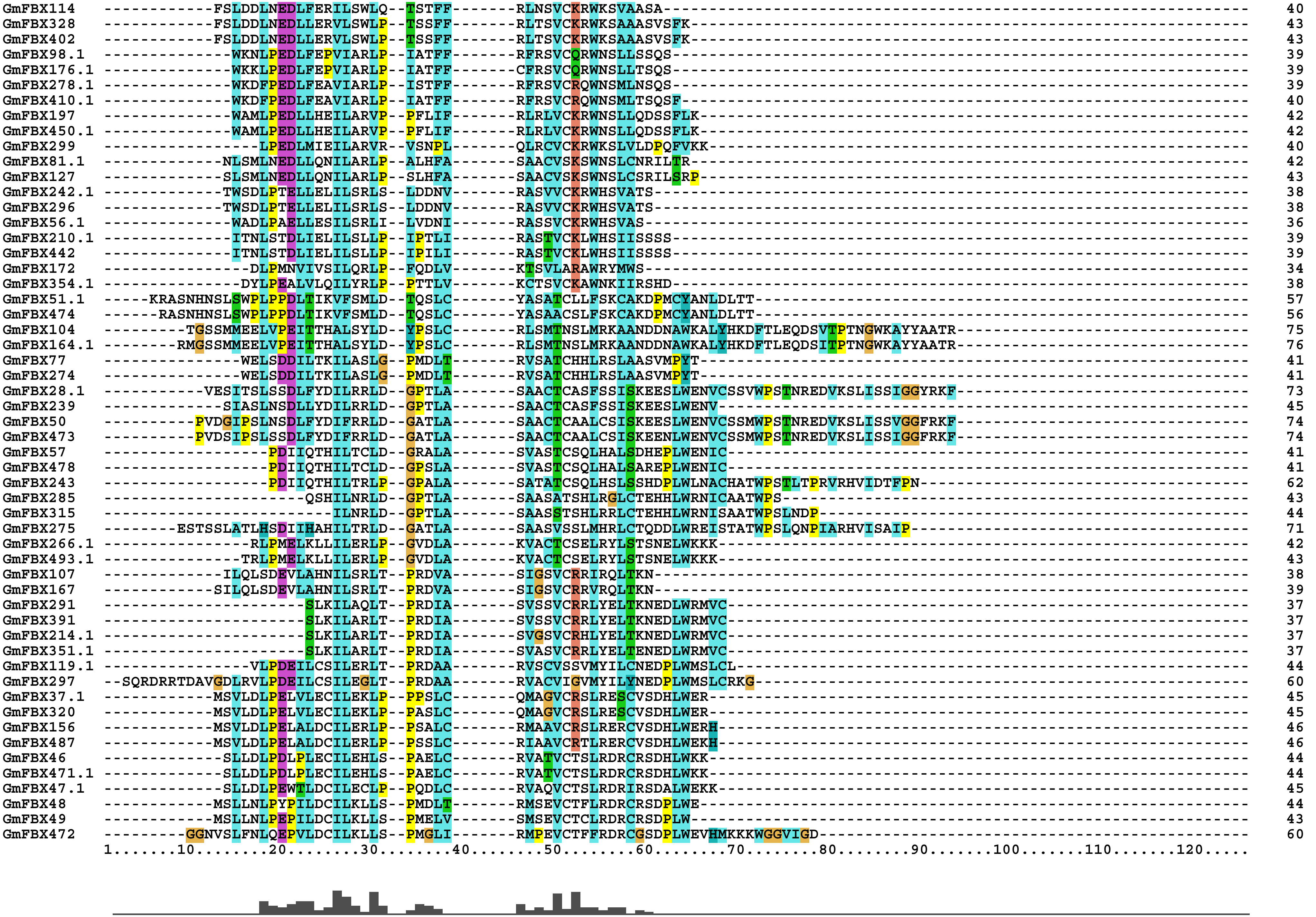
GmFBX114 ------------FSLDDLNEDLFERILSWLQ--TSTFF-------RLNSVCKRWKSVAASA--- 40
GmFBX328 ------------FSLDDLNEDLLERVLSWLP--TSSFF-------RLTSVCKRWKSAAASVSFK-- 43
GmFBX402 ------------FSLDDLNEDLLERVLSWLP--TSSFF-------RLTSVCKRWKSAAASVSFK-- 43
GmFBX98.1 --------------WKNLPEDLFEPVIARLP--IATFF-------RFRSVCQRWNSLLSSQS--- 39
GmFBX176.1 --------------WKKLPEDLFEPVIARLP--IATFF-------CFRSVCQRWNSLLTSQS--- 39
GmFBX278.1 --------------WKDFPEDLFEAVIARLP--ISTFF-------RFRSVCRQWNSMLNSQS--- 39
GmFBX410.1 --------------WKDFPEDLFEAVIARLP--IATFF-------RFRSVCRQWNSMLTSQSF-- 40
GmFBX197 --------------WAMLPEDLLHEILARVP--PFLIF-------RLRLVCKRWNSLLQDSSFLK-- 42
GmFBX450.1 --------------WAMLPEDLLHEILARVP--PFLIF-------RLRLVCKRWNSLLQDSSFLK-- 42
GmFBX299 -----------------LPEDLMIEILARVR--VSNPL-------QLRCVCKRWKSLVLDPQFVKK-- 40
GmFBX81.1 -------------NLSMLNEDLLQNILARLP--ALHFA-------SAACVSKSWNSLCNRILTR--- 42
GmFBX127 -------------SLSMLNEDLLQNILARLP--SLHFA-------SAACVSKSWNSLCSRILSRP-- 43
GmFBX242.1 -------------TWSDLPTELLELILSRLS--LDDNV-------RASVVCKRWHSVATS--- 38
GmFBX296 -------------TWSDLPTELLELILSRLS--LDDNV-------RASVVCKRWHSVATS--- 38
GmFBX56.1 --------------WADLPAELLESILSRLI--LVDNI-------RASSVCKRWHSVAS-- 36
GmFBX210.1 --------------ITNLSTDLIELILSLLP--IPTLI-------RASTVCKLWHSIISSSS--- 39
GmFBX442 --------------ITNLSTDLIELILSLLP--IPILI-------RASTVCKLWHSIISSSS--- 39
GmFBX172 ---------------DLPMNVIVSILQRLP--FQDLV-------KTSVLARAWRYMWS--- 34
GmFBX354.1 ---------------DYLPEALVLQILYRLP--PTTLV-------KCTSVCKAWNKIIRSHD--- 38
GmFBX51.1 -----KRASNHNSLSWPLPPDLTIKVFSMLD--TQSLC-------YASATCLLFSKCAKDPMCYANLDLTT-- 57
GmFBX474 ------RASNHNSLSWPLPPDLTIKVFSMLD--TQSLC-------YASAACSLFSKCAKDPMCYANLDLTT-- 56
GmFBX104 ---------TGSSMMEELVPEITTHALSYLD--YPSLC-------RLSMTNSLMRKAANDDNAWKALYHKDFTLEQDSVTPTNGWKAYYAATR---------------------------------- 75
GmFBX164.1 --------RMGSSMMEELVPEITTHALSYLD--YPSLC-------RLSMTNSLMRKAANDDNAWKALYHKDFTLEQDSITPTNGWKAYYAATR---------------------------------- 76
GmFBX77 ---------------WELSDDILTKILASLG--PMDLT-------RVSATCHHLRSLAASVMPYT--- 41
GmFBX274 ---------------WELSDDILTKILASLG--PMDLT-------RVSATCHHLRSLAASVMPYT--- 41
GmFBX28.1 ------------VESITSLSSDLFYDILRRLD--GPTLA-------SAACTCASFSSISKEESLWENVCSSVWPSTNREDVKSLISSIGGYRKF------------------------------- 73
GmFBX239 -------------SIASLNSDLLYDILRRLD--GPTLA-------SAACTCASFSSISKEESLWENV--- 45
GmFBX50 ----------PVDGIPSLNSDLFYDIFRRLD--GATLA-------SAACTCAALCSISKEESLWENVCSSMWPSTNREDVKSLISSVGGFRKF------------------------------- 74
GmFBX473 ----------PVDSIPSLSSDLFYDIFRRLD--GATLA-------SAACTCAALCSISKEENLWENVCSSMWPSTNREDVKSLISSIGGFRKF------------------------------- 74
GmFBX57 ------------------PDIIQTHILTCLD--GRALA-------SVASTCSQLHALSDHEPLWENIC-- 41
GmFBX478 ------------------PDIIQTHILTCLD--GPSLA-------SVASTCSQLHALSAREPLWENIC-- 41
GmFBX243 ------------------PDIIQTHILTRLP--GPALA-------SATATCSQLHSLSSHDPLWLNACHATWPSTLTPRVRHVIDTFPN-------------------------------- 62
GmFBX285 ----------------------QSHILNRLD--GPTLA-------SAASATSHLRGLCTEHHLWRNICAATWPS-- 43
GmFBX315 -------------------------ILNRLD--GPTLA-------SAASSTSHLRRLCTEHHLWRNISAATWPSLNDP--- 44
GmFBX275 --------ESTSSLATLHSDIIHAHILTRLD--GATLA-------SAASVSSLMHRLCTQDDLWREISTATWPSLQNPIARHVISAIP---------------------------------- 71
GmFBX266.1 ---------------RLPMELKLLILERLP--GVDLA-------KVACTCSELRYLSTSNELWKKK-- 42
GmFBX493.1 ---------------TRLPMELKLLILERLP--GVDLA-------KVACTCSELRYLSTSNELWKKK-- 43
GmFBX107 -------------ILQLSDEVLAHNILSRLT--PRDVA-------SIGSVCRRIRQLTKN--- 38
GmFBX167 ------------SILQLSDEVLAHNILSRLT--PRDVA-------SIGSVCRRVRQLTKN--- 39
GmFBX291 -----------------------SLKILAQLT--PRDIA-------SVSSVCRRLYELTKNEDLWRMVC-- 37
GmFBX391 -----------------------SLKILARLT--PRDIA-------SVSSVCRRLYELTKNEDLWRMVC-- 37
GmFBX214.1 -----------------------SLKILARLT--PRDIA-------SVGSVCRHLYELTKNEDLWRMVC-- 37
GmFBX351.1 -----------------------SLKILARLT--PRDIA-------SVASVCRRLYELTENEDLWRMVC-- 37
GmFBX119.1 ---------------VLPDEILCSILERLT--PRDAA-------RVSCVSSVMYILCNEDPLWMSLCL--- 44
GmFBX297 --SQRDRRTDAVGDLRVLPDEILCSILEGLT--PRDAA-------RVACVIGVMYILYNEDPLWMSLCRKG-- 60
GmFBX37.1 ------------MSVLDLPELVLECILEKLP--PPSLC-------QMAGVCRSLRESCVSDHLWER-- 45
GmFBX320 ------------MSVLDLPELVLECILEKLP--PASLC-------QMAGVCRSLRESCVSDHLWER-- 45
GmFBX156 ------------MSVLDLPELALDCILERLP--PSALC-------RMAAVCRSLRERCVSDHLWERH--- 46
GmFBX487 ------------MSVLDLPELALDCILERLP--PSSLC-------RIAAVCRTLRERCVSDHLWEKH--- 46
GmFBX46 -------------SLLDLPDLPLECILEHLS--PAELC-------RVATVCTSLRDRCRSDHLWKK-- 44
GmFBX471.1 -------------SLLDLPDLPLECILEHLS--PAELC-------RVATVCTSLRDRCRSDHLWKK-- 44
GmFBX47.1 -------------SLLDLPEWTLDCILECLP--PQDLC-------RVAQVCTSLRDRIRSDALWEKK--- 45
GmFBX48 ------------MSLLNLPYPILDCILKLLS--PMDLT-------RMSEVCTFLRDRCRSDPLWE--- 44
GmFBX49 ------------MSLLNLPEPILDCILKLLS--PMELV-------SMSEVCTCLRDRCRSDPLW-- 43
GmFBX472 ---------GGNVSLFNLQEPVLDCILKLLS--PMGLI-------RMPEVCTFFRDRCGSDPLWEVHMKKKWGGVIGD-- 60
1.......10.......20.......30.......40.......50.......60.......70.......80.......90.......100.......110.......120.....

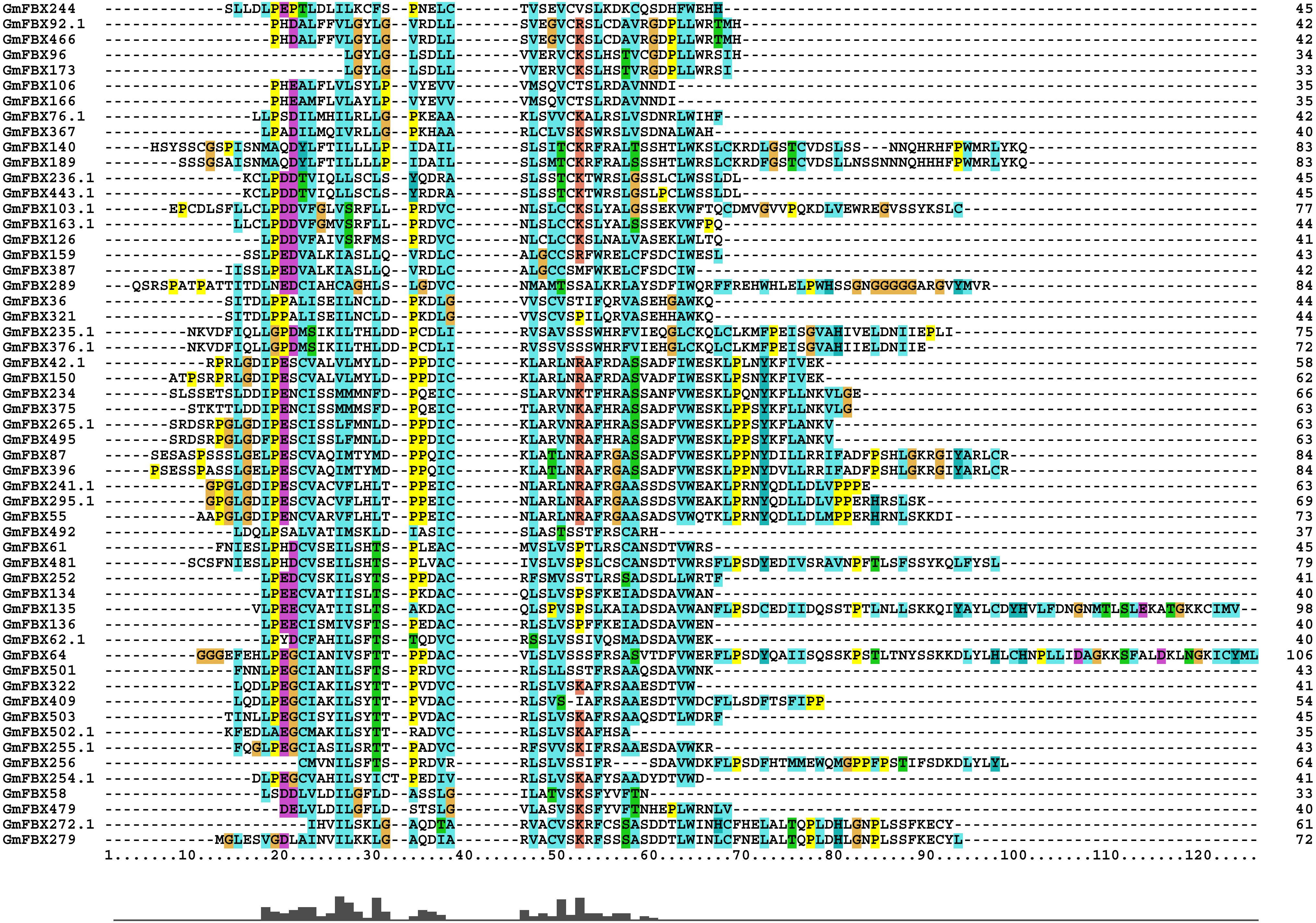
GmFBX244 -------------SLLDLPEPTLDLILKCFS--PNELC-------TVSEVCVSLKDKCQSDHFWEHH-- 45
GmFBX92.1 ------------------PHDALFFVLGYLG--VRDLL-------SVEGVCRSLCDAVRGDPLLWRTMH-- 42
GmFBX466 ------------------PHDALFFVLGYLG--VRDLL-------SVEGVCKSLCDAVRGDPLLWRTMH-- 42
GmFBX96 --------------------------LGYLG--LSDLL-------VVERVCKSLHSTVCGDPLLWRSIH-- 34
GmFBX173 --------------------------LGYLG--LSDLL-------VVERVCKSLHSTVRGDPLLWRSI--- 33
GmFBX106 ------------------PHEALFLVLSYLP--VYEVV-------VMSQVCTSLRDAVNNDI--- 35
GmFBX166 ------------------PHEAMFLVLAYLP--VYEVV-------VMSQVCTSLRDAVNNDI--- 35
GmFBX76.1 ----------------LLPSDILMHILRLLG--PKEAA-------KLSVVCKALRSLVSDNRLWIHF-- 42
GmFBX367 -----------------LPADILMQIVRLLG--PKHAA-------RLCLVSKSWRSLVSDNALWAH--- 40
GmFBX140 -----HSYSSCGSPISNMAQDYLFTILLLLP--IDAIL-------SLSITCKRFRALTSSHTLWKSLCKRDLGSTCVDSLSS---NNQHRHFPWMRLYKQ------------------------- 83
GmFBX189 --------SSSGSAISNMAQDYLFTILLLLP--IDAIL-------SLSMTCKRFRALSSSHTLWRSLCKRDFGSTCVDSLLNSSNNNQHHHFPWMRLYKQ------------------------- 83
GmFBX236.1 ---------------KCLPDDTVIQLLSCLS--YQDRA-------SLSSTCKTWRSLGSSLCLWSSLDL-- 45
GmFBX443.1 ---------------KCLPDDTVIQLLSCLS--YRDRA-------SLSSTCKTWRSLGSLPCLWSSLDL-- 45
GmFBX103.1 -------EPCDLSFLLCLPDDVFGLVSRFLL--PRDVC-------NLSLCCKSLYALGSSEKVWFTQCDMVGVVPQKDLVEWREGVSSYKSLC-------------------------------- 77
GmFBX163.1 --------------LLCLPDDVFGMVSRFLL--PRDVC-------NLSLCCKSLYALSSSEKVWFPQ-- 44
GmFBX126 -----------------LPDDVFAIVSRFMS--PRDVC-------NLCLCCKSLNALVASEKLWLTQ-- 41
GmFBX159 ----------------SSLPEDVALKIASLLQ-VRDLC-------ALGCCSRFWRELCFSDCIWESL-- 43
GmFBX387 --------------IISSLPEDVALKIASLLQ-VRDLC-------ALGCCSMFWKELCFSDCIW--- 42
GmFBX289 ---QSRSPATPATTITDLNEDCIAHCAGHLS--LGDVC-------NMAMTSSALKRLAYSDFIWQRFFREHWHLELPWHSSGNGGGGGARGVYMVR----------------------------- 84
GmFBX36 -------------SITDLPPALISEILNCLD--PKDLG-------VVSCVSTIFQRVASEHGAWKQ--- 44
GmFBX321 -------------SITDLPPALISEILNCLD--PKDLG-------VVSCVSPILQRVASEHHAWKQ--- 44
GmFBX235.1 ---------NKVDFIQLLGPDMSIKILTHLDD-PCDLI-------RVSAVSSSWHRFVIEQGLCKQLCLKMFPEISGVAHIVELDNIIEPLI--------------------------------- 75
GmFBX376.1 ---------NKVDFIQLLGPDMSIKILTHLDD-PCDLI-------RVSSVSSSWHRFVIEHGLCKQLCLKMFPEISGVAHIIELDNIIE------------------------------------ 72
GmFBX42.1 -----------RPRLGDIPESCVALVLMYLD--PPDIC-------KLARLNRAFRDASSADFIWESKLPLNYKFIVEK--- 58
GmFBX150 -------ATPSRPRLGDIPESCVALVLMYLD--PPDIC-------KLARLNRAFRDASVADFIWESKLPSNYKFIVEK--- 62
GmFBX234 -------SLSSETSLDDIPENCISSMMMNFD--PQEIC-------SLARVNKTFHRASSANFVWESKLPQNYKFLLNKVLGE--- 66
GmFBX375 ---------STKTTLDDIPENCISSMMMSFD--PQEIC-------TLARVNKAFHRASSADFVWESKLPPSYKFLLNKVLG-- 63
GmFBX265.1 -------SRDSRPGLGDIPESCISSLFMNLD--PPDIC-------KLARVNRAFHRASSADFVWESKLPPSYKFLANKV-- 63
GmFBX495 -------SRDSRPGLGDFPESCISSLFMNLD--PPDIC-------KLARVNRAFHRASSADFVWESKLPPSYKFLANKV-- 63
GmFBX87 -----SESASPSSSLGELPESCVAQIMTYMD--PPQIC-------KLATLNRAFRGASSADFVWESKLPPNYDILLRRIFADFPSHLGKRGIYARLCR--------------------------- 84
GmFBX396 -----PSESSPASSLGELPESCVAQIMTYMD--PPQIC-------KLATLNRAFRGASSADFVWESKLPPNYDVLLRRIFADFPSHLGKRGIYARLCR--------------------------- 84
GmFBX241.1 -----------GPGLGDIPESCVACVFLHLT--PPEIC-------NLARLNRAFRGAASSDSVWEAKLPRNYQDLLDLVPPPE-- 63
GmFBX295.1 -----------GPGLGDIPESCVACVFLHLT--PPEIC-------NLARLNRAFRGAASSDSVWEAKLPRNYQDLLDLVPPERHRSLSK------------------------------------ 69
GmFBX55 ----------AAPGLGDIPENCVARVFLHLT--PPEIC-------NLARLNRAFRGAASADSVWQTKLPRNYQDLLDLMPPERHRNLSKKDI--------------------------------- 73
GmFBX492 --------------LDQLPSALVATIMSKLD--IASIC-------SLASTSSTFRSCARH--- 37
GmFBX61 -------------FNIESLPHDCVSEILSHTS-PLEAC-------MVSLVSPTLRSCANSDTVWRS--- 45
GmFBX481 ----------SCSFNIESLPHDCVSEILSHTS-PLVAC-------IVSLVSPSLCSCANSDTVWRSFLPSDYEDIVSRAVNPFTLSFSSYKQLFYSL---------------------------- 79
GmFBX252 -----------------LPEDCVSKILSYTS--PPDAC-------RFSMVSSTLRSSADSDLLWRTF-- 41
GmFBX134 -----------------LPEECVATIISLTS--PKDAC-------QLSLVSPSFKEIADSDAVWAN--- 40
GmFBX135 ----------------VLPEECVATIISLTS--AKDAC-------QLSPVSPSLKAIADSDAVWANFLPSDCEDIIDQSSTPTLNLLSKKQIYAYLCDYHVLFDNGNMTLSLEKATGKKCIMV-- 98
GmFBX136 -----------------LPEECISMIVSFTS--PEDAC-------RLSLVSPFFKEIADSDAVWEN--- 40
GmFBX62.1 -----------------LPYDCFAHILSFTS--TQDVC-------RSSLVSSIVQSMADSDAVWEK--- 40
GmFBX64 ----------GGGEFEHLPEGCIANIVSFTT--PPDAC-------VLSLVSSSFRSASVTDFVWERFLPSDYQAIISQSSKPSTLTNYSSKKDLYLHLCHNPLLIDAGKKSFALDKLNGKICYML 106
GmFBX501 --------------FNNLPEGCIANILSFTS--PRDVC-------RLSLLSSTFRSAAQSDAVWNK--- 43
GmFBX322 --------------LQDLPEGCIAKILSYTT--PVDVC-------RLSLVSKAFRSAAESDTVW--- 41
GmFBX409 --------------LQDLPEGCIAKILSYTT--PVDAC-------RLSVS-IAFRSAAESDTVWDCFLLSDFTSFIPP--- 54
GmFBX503 -------------TINLLPEGCISYILSYTT--PVDAC-------RLSLVSKAFRSAAQSDTLWDRF-- 45
GmFBX502.1 -------------KFEDLAEGCMAKILSYTT--RADVC-------RLSLVSKAFHSA-- 35
GmFBX255.1 --------------FQGLPEGCIASILSRTT--PADVC-------RFSVVSKIFRSAAESDAVWKR--- 43
GmFBX256 ---------------------CMVNILSFTS--PRDVR-------RLSLVSSIFR----SDAVWDKFLPSDFHTMMEWQMGPPFPSTIFSDKDLYLYL--------------------------- 64
GmFBX254.1 ----------------DLPEGCVAHILSYICT-PEDIV-------RLSLVSKAFYSAADYDTVWD-- 41
GmFBX58 ------------------LSDDLVLDILGFLD-ASSLG-------ILATVSKSFYVFTN-- 33
GmFBX479 -------------------DELVLDILGFLD--STSLG-------VLASVSKSFYVFTNHEPLWRNLV--- 40
GmFBX272.1 ----------------------IHVILSKLG--AQDTA-------RVACVSKRFCSSASDDTLWINHCFHELALTQPLDHLGNPLSSFKECY--------------------------------- 61
GmFBX279 ------------MGLESVGDLAINVILKKLG--AQDIA-------RVACVSKRFSSSASDDTLWINLCFNELALTQPLDHLGNPLSSFKECYL-------------------------------- 72
1.......10.......20.......30.......40.......50.......60.......70.......80.......90.......100......110......120.....

```
GmFBX404    -------------SFTDFPEDIQVCILSFLG--PPEIA-------TLACTSKRMG----------SLCATDSK----------------------------------------------------   41
GmFBX509    -------------SFTDFPEDIQVSILSFLG--PSEIA-------TLACTSKKLG----------SLCSTVS-----------------------------------------------------   40
GmFBX362    -----------HSTFSWFEEDIWTEIAKFLD--GKSLV-------MLAATSRWFRRAIMDDGIWKFVCLRDLQVPPPECVAFRWCKL--------------------------------------   67
GmFBX9.1    ---------------LRLSDDCLAAIFHFLNT-ADRK--------RCSLVCLRWRLVDGQRRHRL------------------------------------------------------------   41
GmFBX268    ---------------LRLSDDCLAAIFHFLST-ADRK--------RCSLVCRRWLRVDGQRRHRL------------------------------------------------------------   41
GmFBX93     ---------------ADIPDECLAGIFQFLSS-VDRK--------TCSAVCRRWLR---------------------------------------------------------------------   32
GmFBX398    ---------------ADIPDECLAGIFQFLSS-VDRK--------TCSAVCRRWLR---------------------------------------------------------------------   32
GmFBX324    --------------ISDLPNECLASVFQFLSS-ADRN--------RCSLVCRRWLQIE-------------------------------------------------------------------   35
GmFBX406    --------------ISDLPNECLASVFQFLSS-ADRS--------RCSLVCRRWL----------------------------------------------------------------------   32
GmFBX68     --------------IDDIPDNCLASIFQLFPP-VDHK--------NFSLVCRRC-----------------------------------------------------------------------   31
GmFBX110    --------------IDDIPDNCLACIFQLFPP-ADQK--------KLSLVCRRWLKVEGHTHHRLC-----------------------------------------------------------   43
GmFBX363    ----------------YFPDEVIEHIFDYVVS-HSDR-------NALSLVCKSWYR---------------------------------------------------------------------   32
GmFBX469    ----------------YFPDEVIEHIFDYVVS-HSDR-------NALSLVCKSWYR---------------------------------------------------------------------   32
GmFBX18     ------------------PDEVIEHIFDYVTS-QRDR-------NDLSLVCKNWHR---------------------------------------------------------------------   30
GmFBX30.1   ------------------PEEVLEHVFSFIEC-DKDR-------GSISLVCKSWYE---------------------------------------------------------------------   30
GmFBX238.1  ------------------PEEVLEHVFSFIEC-DKDR-------GSISLVCKSWYE---------------------------------------------------------------------   30
GmFBX59     ------------------PEEVLEHVFSFIWS-ERDR-------NAISLVCKSWYE---------------------------------------------------------------------   30
GmFBX480    ------------------PEEVLEHVFSFIWN-ERDR-------NAISLVCKSWYE---------------------------------------------------------------------   30
GmFBX34     -------------FPDQVLENVLENVLHFLPS-RRDR-------NAASLVCRSWYR---------------------------------------------------------------------   35
GmFBX333    -------------FPDQVLENVLENVLHFLSS-RRDR-------NAASLVCRSWYR---------------------------------------------------------------------   35
GmFBX74     --------RTLSPLPDQVLENVLESVLHFLTS-RRDR-------NAASLVCKSWYR---------------------------------------------------------------------   40
GmFBX117    ---------SLSPLPEQVLENVLESVLHFLTS-RRDR-------NAASLVCKSWYR---------------------------------------------------------------------   39
GmFBX142    ---------------KDLPEECWELVFRFIGH-GRHL-------ESLSLVCKQFLSITNRLQFS-------------------------------------------------------------   41
GmFBX73     -------------SIESLPDECLFEILRRLPA-GQDR-------SVCASVSKRWLMLLSSICKN-------------------------------------------------------------   43
GmFBX115    -------------SIESLPDECLFEILRRLPA-GQDR-------SVCASVSKRWLMLLSSI----------------------------------------------------------------   40
GmFBX329    -------------SIEALPDECLFEIFRRLPA-GEDR-------SACACVSKRWLMLLSSI----------------------------------------------------------------   40
GmFBX401    ---------------EALPDECLFEIFRRLPS-GEDR-------SACACVSKRWLM---------------------------------------------------------------------   33
GmFBX298    ---------------EVLPDECLFEIFRRLPS-GKER-------SSCACVSKRW-----------------------------------------------------------------------   31
GmFBX393.1  ---------------EVLPDECLFEIFRRLPS-GKER-------SSCACVSKRW-----------------------------------------------------------------------   31
GmFBX78     ---------------DVLPDECLFEVFRWLSS-GKER-------SSCAYVSKRW-----------------------------------------------------------------------   31
GmFBX161    ------------------PDDLIVEIFSRLHS-MSTR-------DACSLVCRRWFR---------------------------------------------------------------------   30
GmFBX384    ------------------PDELIVEIFSRLHS-KSTR-------DACSLVCRRWFR---------------------------------------------------------------------   30
GmFBX301.1  -----------------LPDELLIEIFRRLDS-KSNR-------DASSLVCTRWLR---------------------------------------------------------------------   31
GmFBX346    -----------------IPDELLIEIFRRLDS-KSSR-------DAGSLVCTRWLR---------------------------------------------------------------------   31
GmFBX145    ---------NDCTSIMHLPDDCLVIIFHGLDS-RIDR-------DSFGLTCRRWLH---------------------------------------------------------------------   39
GmFBX273    ----------------------LMHIFNFLPP-IPDR-------FNTALVCHRWNYLACHPRLWLRV----------------------------------------------------------   37
GmFBX334.1  --------------MDNIPEHIVWEILSRLKK-TSDR-------NSVSLVCKRLYYLDNAQRTFVRVGC--------------------------------------------------------   47
GmFBX366    -------------------DELLQEIFQKLPS-SSS--------SSVSLVCKRWLRL--------------------------------------------------------------------   29
GmFBX465    -------------------DELLQEILQKLPS-SSS--------SSVSLVCKRWLRL--------------------------------------------------------------------   29
GmFBX137    -------------------DELLLRILSKLPD-SSQQ------RNSNSLVCKRWLN---------------------------------------------------------------------   30
GmFBX470.1  -------------CWLLLPDELWRRILEIGIE-SNGFSY--KDLCCVSISCRRLHRLSSEEPLWNRLLFSDYPQSQSHPSSSSSSSSSKSLYRLR------------------------------   79
GmFBX158    -GSNKKHGSVSVTTIKSLPKELQVEIFAKVAT-RSI-----FDHCMIKLCCKEFLHAAEDDYVYRHASMENFALVPLPWF---------------------------------------------   73
GmFBX388    ----KKHGSVSVTAIKFLPKELQVEIFAKVAT-RSV-----FDHCMIKLCCKEFLRAAEDNYVYRHASMENFALVPLPW----------------------------------------------   69
GmFBX35     ---------DDKPPILSLPSDLLEEIIAKAAS-KSS-----IDLVNIKLSCKDFLHASEANNVWKNVSLEDFPSGWYPH----------------------------------------------   64
GmFBX80     ---------KKHDLFECLPDDLLVLILSKLSS-TASSP---SDFINIILTCKRLNRLGLHRLVLSKAASKLFAI---------------------------------------------------   61
GmFBX128    -------SKNKHDLFECLPDDLVVLILSKLSA-TASSP---SDFINIILTCKRLNRSGLHRLVL-------------------------------------------------------------   53
GmFBX97     -KTPEKTAGADYEFFESLPDDLVISIFCKLSS-TATKP---SDFVNILITCKRLNRLALHSLVLSKASPKTFTI---------------------------------------------------   69
GmFBX175    ----EKLAAGRYDFFESLPDDLVISIFCKLSS-TATKP---SDFVNVLITSKRLNRLALHSLVLSKASPRTFTI---------------------------------------------------   66
GmFBX304.1  ---------------DRLPDSLLLLVFNKIG-----------DVKALGRCCVVSRRFHSL-----------------------------------------------------------------   34
GmFBX343.1  ---------------DRLPDSLLLLVFNKIG-----------DVKALGRCCVVSRRFHSL-----------------------------------------------------------------   34
GmFBX8      --------------IDRLPIDLLAHIFVLFTS-FTDLA-------QASGVCK---------KWKQGVKESLAR----------------------------------------------------   42
GmFBX269.1  --------------IDRLPIDLLAHIFVLFTS-FTDLA-------QASGVCK---------KWKQGVKESLAR----------------------------------------------------   42
GmFBX44     --------------IDRLPIDLLAYIFVLFTS-FTDLA-------QASGVCK---------KWKQGVKESLAR----------------------------------------------------   42
GmFBX102.1  ---------------DSMPDAILQCILSRITN-ARDVS-------SCNCVSK---------RWKDS-----------------------------------------------------------   34
GmFBX178    ---------------DSLPDAILQCILSRITN-ARDVS-------SCNCVSK---------RWKDS-----------------------------------------------------------   34
            1......10........20........30........40........50........60........70........80........90......100......110......120....
```

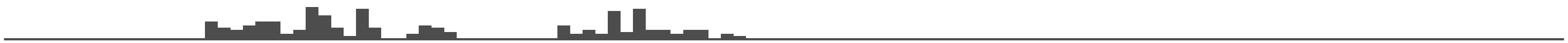

```
GmFBX182    -----------------LPDAILQYILSHINN-ARDVA-------ACNCVSK---------RWKD------------------------------------------------------------    31
GmFBX337    -----------------LPDAILQYILSRINN-ARDVA-------ACNCVSK---------RWKD------------------------------------------------------------    31
GmFBX165    ---------IAMGTMEDLPASVLAEILSRFTD-TTDVA-------RCRVVSKSLNAASYEVRWLNLV----------------------------------------------------------    50
GmFBX276.1  ---------------DQLPVEVIGNILSHLRA-ARDVV-------IASATCR---------KWRQACCK--------------------------------------------------------    37
GmFBX281    ---------------DQLPVEVIGNILSHLRA-ARDVV-------IASASCR---------KWRLACCK--------------------------------------------------------    37
GmFBX477    ---------------EHLPVEVIGNILSLLGS-ARDVV-------VASVTCK---------KWREA-----------------------------------------------------------    34
GmFBX264.1  --------------------DILVKIFQLLDI-FELTS-------GISRVCS---------AWRLACCDPLLWKTLD------------------------------------------------    40
GmFBX496.1  --------------------DILVKIFQLLDI-FELTS-------GISRVCS---------AWRMACCDPLLWKTLD------------------------------------------------    40
GmFBX305    --------------WLDLPRDVLCTIFQKLGA-TEILT-------RAQGVCS---------VWRAISKEPLLWRTID------------------------------------------------    46
GmFBX341    --------------WLDLPRDVVCTIFQKLGA-IEILT-------RAQRVCS---------VWRGISKEPLLWRTID------------------------------------------------    46
GmFBX192.1  -------SASCQCTKGHLSEDVIFLVFHNLNWNPKLIA-------TLSCVCKWFDDLAKRVLWKEFCRTRAPKMLCDLQ----------------------------------------------    65
GmFBX444.1  -------SASCQCTKGHLSEDMIFLVFHNLNWNPKLIA-------TLSCVCKWFDDLAKRVLWKEFCRTRAPKMLCDLQ----------------------------------------------    65
GmFBX288.1  -------SASCQCTKGHLSEDVIFLVFQHLNWNPKLIA-------ALSCVCKWFDDLSKQVLWKEFCKTRAPKMMLDLQSSGSHSVDGN------------------------------------    75
GmFBX454    ---------------GHVSEDVIFLVFQHLNWNPKLIA-------ALSCVCKWFDDLSKQVLWKEFCKTRAPKMMLDLQSSGSHSVDGN------------------------------------    67
GmFBX3.1    ---------MGPDLISDLPQSIIESILVQLP--IRDA-----VRTS-ILSSKWRYKWASITRLVFDDKCVPF-----------------------------------------------------    55
GmFBX25     ---------MGPDLISDLPQSIIESILVQLP--IRDA-----VRTS-ILSSKWRYKWASITQLVFDDKCVPFS----------------------------------------------------    56
GmFBX23     -------------RISDLPSHLIDFILQRLP--LQDV-----VRTS-LLSSKWRYKWTSI-----------------------------------------------------------------    39
GmFBX24     -------------RISDLPSHLIDFILQRLQ--LQDV-----VRTS-LLSSKWRYKWTS------------------------------------------------------------------    38
GmFBX407    -------------RISCLPGHVIDQVLSHLS--IREA-----VRTS-VLSSKWR-----------------------------------------------------------------------    33
GmFBX336.1  ----------SVDRISQFPDHVIHHILSHLRN-VNDA-----IRTS-VLSKRWRELWYSYSVL--------------------------------------------------------------    46
GmFBX184.1  --------------IENLPDIVLHDILSRLP--EKDA-----ARTS-VLSKKWAEIWS-------------------------------------------------------------------    36
GmFBX185.1  --------------ISTLPKTILHDILSRMP--EEDA-----VRTS-VLSKSWAET---------------------------------------------------------------------    34
GmFBX280    ----------NRDIISTMPDDILGDILSRLT--LKEA-----ARTS-VLATKWRYHWTFFSGLLDFDHSLRNF----------------------------------------------------    55
GmFBX395    -----------GDLINKLPDGIPVAILSKLP--INEA-----ARTS-ILSRKWRYLW--------------------------------------------------------------------    38
GmFBX248    -------------RLSELPDFVLLHIMNFID--TKDA-----LRTC-ILSKRWKDLWK-------------------------------------------------------------------    37
GmFBX250    -------------RLSELPDFVLLHIMNFID--TKDA-----LRTC-ILSKRWKDLWK-------------------------------------------------------------------    37
GmFBX249    ----------ERDRLSELPDFVLLHIMNFIY--TKDA-----LRTC-ILSKRWKDLWKHLTTLSFYQS---------------------------------------------------------    50
GmFBX373    -------------RISELPDNVLLHIMNFVD--TKDA-----VKTC-VLSKRWKDLGKG------------------------------------------------------------------    38
GmFBX17     -------------RISELPDCILMHIMSFLD--TKDA-----VQTC-ILSKRWKDLC--------------------------------------------------------------------    36
GmFBX220    -------------NISEFPDHVLLHIMSLMD--TKSA-----VRTC-VLSKRWKDLCKRL-----------------------------------------------------------------    39
GmFBX219    -------------RISELPDSILLHILNFMN--TESA-----VQTC-VLSKRWKDLCKR------------------------------------------------------------------    38
GmFBX224    -------------RISELPISVLLHILEFMN--TKDA-----VQTC-VLSKPWKDLCKR------------------------------------------------------------------    38
GmFBX225    -------------RISELPDSVLLHIIEFMD--TKSG-----VQTC-VLSKRWKDLWKS------------------------------------------------------------------    38
GmFBX306.1  ----------RPDRISALPDSLLFHMINFMD--TKSA-----VQTC-VLSKRWNDLSKCLTNLTFNS----------------------------------------------------------    49
GmFBX233.1  -------------RLSDLPDFVLLHIMKFMS--MKHA-----VQTC-VLSKRWKELWKR------------------------------------------------------------------    38
GmFBX374    -------------RLSDLPDLVLLHIMKFMS--MKHA-----VQTC-VLSTRWKELWKRL-----------------------------------------------------------------    39
GmFBX105    --------------LSDLPECILLHIMKFMN--TRHA-----VQTC-VLSKRWKDLWKR------------------------------------------------------------------    37
GmFBX226.1  -------------RLSELPDCVVLHIMEFMD--TKYA-----VQTC-VLSKRWKDLWKR------------------------------------------------------------------    38
GmFBX227.1  -------------RLSELPDCVVLHIMEFMD--TKYA-----VQTC-VLSKRWKDLWKR------------------------------------------------------------------    38
GmFBX228.1  -------------RLSELPDCVVLHIMEFMD--TKYA-----VQTC-VLSKRWKDLWKR------------------------------------------------------------------    38
GmFBX229.1  -------------RLSELPDCVVLHIMEFMD--TKYA-----VQTC-VLSKRWKDLWKR------------------------------------------------------------------    38
GmFBX230.1  -------------RLSELPDCVVLHIMEFMD--TKYA-----VQTC-VLSKRWKDLWKR------------------------------------------------------------------    38
GmFBX231.1  -------------RLSELPDCVVLHIMEFMD--TKYA-----VQTC-VLSKRWKDLWKR------------------------------------------------------------------    38
GmFBX232    -------------RLSELPDCVVLHIMEFMD--TKYA-----VQTC-VLSKRWKDLWKR------------------------------------------------------------------    38
GmFBX247    -------------RLSELPDCILLYIMKFMN--TKYA-----VQTC-ILSKRWKNLWKR------------------------------------------------------------------    38
GmFBX485    -------------RLSELPDSVLVHIMELME--TRNA-----VQTC-VLSQRWKNLWRR------------------------------------------------------------------    38
GmFBX221    -------------KISEMPDNILLHMMNFMD--TREA-----VQTC-VLSKRWNNLWKR------------------------------------------------------------------    38
GmFBX222    -------------KISELPDNILLHMMNFMD--TREA-----VQTC-VLSKRWNNLWKR------------------------------------------------------------------    38
GmFBX223    -------------KISELPDNILLHMMDFMD--TREA-----VQTC-VLSKRWNNLWKRL-----------------------------------------------------------------    39
GmFBX251.1  ----------------KLPENVLLHIMNFME--TRHA-----VQTC-VLSKRWNNLWKS------------------------------------------------------------------    35
GmFBX218    -------------RISELPDTVLLHILNFMN--TKDA-----VKTS-VLFNRWKNFCKAL-----------------------------------------------------------------    39
GmFBX118.1  -------------RLSDLPECVLLHILTFLN--AKHA-----VRTC-VLSTRWKDLWKR------------------------------------------------------------------    38
GmFBX355    -------------RISELPIHVFLRILEFMN--TRDA-----VRLC-ALSKSWKDFWKR------------------------------------------------------------------    38
GmFBX40     -------------RLSNLPDEVLHRILSSLD--AKSA-----VQTC-VLSKRWTHVWTS------------------------------------------------------------------    38
GmFBX138    --------------VSNLPDEVLHRILSTLD--AKSA-----VQTC-VLSKRWRHVWTS------------------------------------------------------------------    37
            1.......10........20........30........40........50........60........70........80........90.......100.......110.......120.....
```

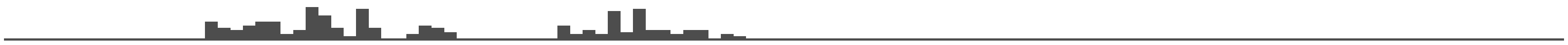

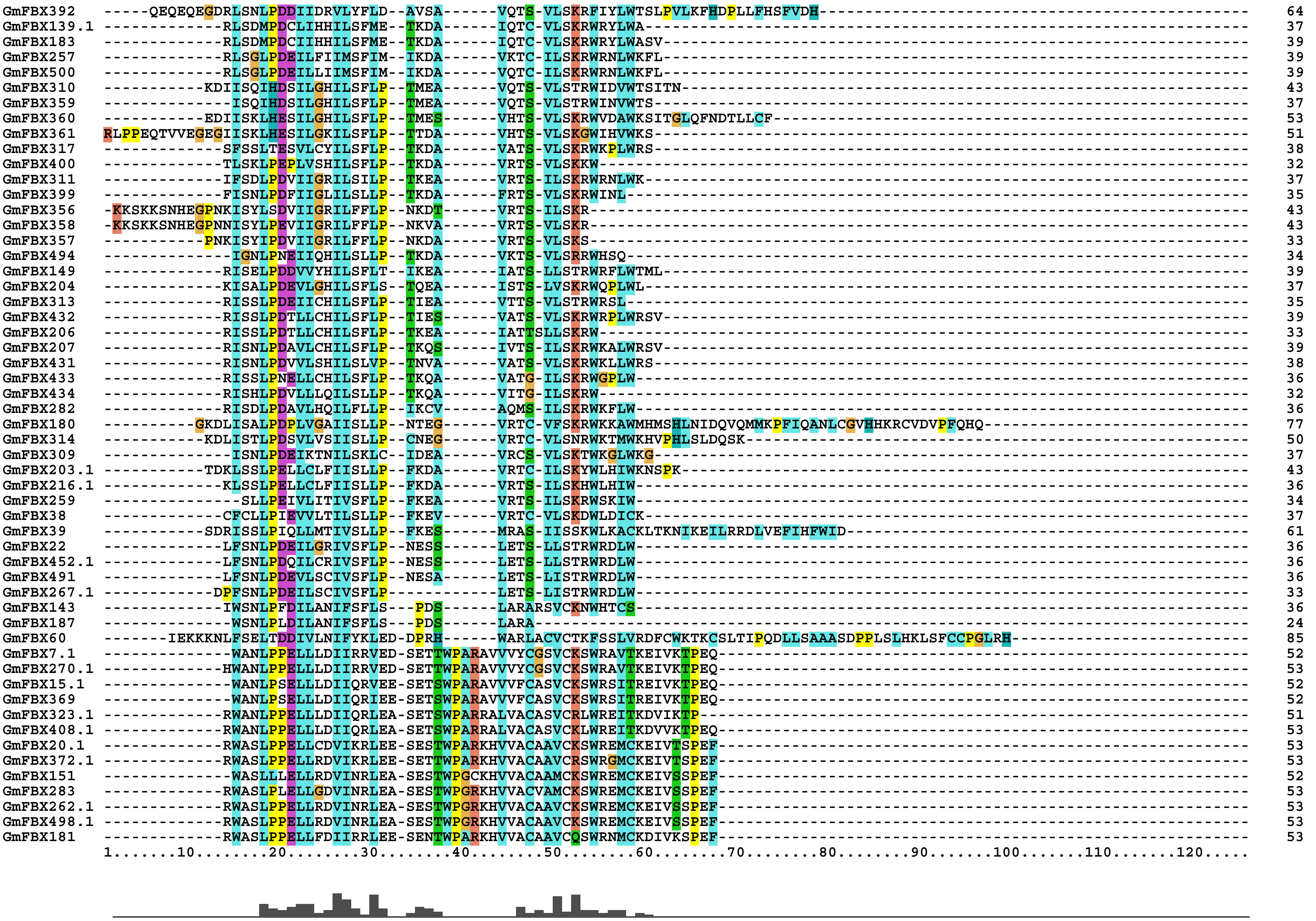
GmFBX392 -----QEQEQEGDRLSNLPDDIIDRVLYFLD--AVSA------VQTS-VLSKRFIYLWTSLPVLKFHDPLLFHSFVDH--- 64
GmFBX139.1 -------------RLSDMPDCLIHHILSFME--TKDA------IQTC-VLSKRWRYLWA-- 37
GmFBX183 -------------RLSDMPDCIIHHILSFME--TKDA------IQTC-VLSKRWRYLWASV-- 39
GmFBX257 -------------RLSGLPDEILFIIMSFIM--IKDA------VKTC-ILSKRWRNLWKFL-- 39
GmFBX500 -------------RLSGLPDEILLIIMSFIM--IKDA------VQTC-ILSKRWRNLWKFL-- 39
GmFBX310 -----------KDIISQIHDSILGHILSFLP--TMEA------VQTS-VLSTRWIDVWTSITN-- 43
GmFBX359 --------------ISQIHDSILGHILSFLP--TMEA------VQTS-VLSTRWINVWTS--- 37
GmFBX360 -----------EDIISKLHESILGHILSFLP--TMES------VHTS-VLSKRWVDAWKSITGLQFNDTLLCF-- 53
GmFBX361 RLPPEQTVVEGEGIISKLHESILGKILSFLP--TTDA------VHTS-VLSKGWIHVWKS--- 51
GmFBX317 -------------SFSSLTESVLCYILSFLP--TKDA------VATS-VLSKRWKPLWRS--- 38
GmFBX400 -------------TLSKLPEPLVSHILSFLP--TKDA------VRTS-VLSKKW--- 32
GmFBX311 -------------IFSDLPDVIIGRILSILP--TKEA------VRTS-ILSKRWRNLWK-- 37
GmFBX399 -------------FISNLPDFIIGLILSLLP--TKDA------FRTS-VLSKRWINL-- 35
GmFBX356 -KKSKKSNHEGPNKISYLSDVIIGRILFFLP--NKDT------VRTS-ILSKR-- 43
GmFBX358 -KKSKKSNHEGPNNISYLPEVIIGRILFFLP--NKVA------VRTS-VLSKR-- 43
GmFBX357 -----------PNKISYIPDVIIGRILFFLP--NKDA------VRTS-VLSKS-- 33
GmFBX494 --------------IGNLPNEIIQHILSLLP--TKDA------VKTS-VLSRRWHSQ-- 34
GmFBX149 -------------RISELPDDVVYHILSFLT--IKEA------IATS-LLSTRWRFLWTML-- 39
GmFBX204 -------------KISALPDEVLGHILSFLS--TQEA------ISTS-LVSKRWQPLWL-- 37
GmFBX313 -------------RISSLPDEIICHILSFLP--TIEA------VTTS-VLSTRWRSL-- 35
GmFBX432 -------------RISSLPDTLLCHILSFLP--TIES------VATS-VLSKRWRPLWRSV-- 39
GmFBX206 -------------RISSLPDTLLCHILSFLP--TKEA------IATTSLLSKRW--- 33
GmFBX207 -------------RISNLPDAVLCHILSFLP--TKQS------IVTS-ILSKRWKALWRSV-- 39
GmFBX431 -------------RISNLPDVVLSHILSLVP--TNVA------VATS-VLSKRWKLLWRS--- 38
GmFBX433 -------------RISSLPNELLCHILSFLP--TKQA------VATG-ILSKRWGPLW--- 36
GmFBX434 -------------RISHLPDVLLLQILSLLP--TKQA------VITG-ILSKRW--- 32
GmFBX282 -------------RISDLPDAVLHQILFLLP--IKCV------AQMS-ILSKRWKFLW--- 36
GmFBX180 ----------GKDLISALPDPLVGAIISLLP--NTEG------VRTC-VFSKRWKKAWMHMSHLNIDQVQMMKPFIQANLCGVHHKRCVDVPFQHQ----------------------------- 77
GmFBX314 -----------KDLISTLPDSVLVSIISLLP--CNEG------VRTC-VLSNRWKTMWKHVPHLSLDQSK--- 50
GmFBX309 --------------ISNLPDEIKTNILSKLC--IDEA------VRCS-VLSKTWKGLWKG--- 37
GmFBX203.1 -----------TDKLSSLPELLCLFIISLLP--FKDA------VRTC-ILSKYWLHIWKNSPK-- 43
GmFBX216.1 -------------KLSSLPELLCLFIISLLP--FKDA------VRTS-ILSKHWLHIW--- 36
GmFBX259 ---------------SLLPEIVLITIVSFLP--FKEA------VRTS-ILSKRWSKIW--- 34
GmFBX38 -------------CFCLLPIEVVLTILSLLP--FKEV------VRTC-VLSKDWLDICK-- 37
GmFBX39 -----------SDRISSLPIQLLMTIVSLLP--FKES------MRAS-IISSKWLKACKLTKNIKEILRRDLVEFIHFWID-- 61
GmFBX22 -------------LFSNLPDEILGRIVSFLP--NESS------LETS-LLSTRWRDLW--- 36
GmFBX452.1 -------------LFSNLPDQILCRIVSFLP--NESS------LETS-LLSTRWRDLW--- 36
GmFBX491 -------------LFSNLPDEVLSCIVSFLP--NESA------LETS-LISTRWRDLW--- 36
GmFBX267.1 ------------DPFSNLPDEILSCIVSFLP------------LETS-LISTRWRDLW--- 33
GmFBX143 -------------IWSNLPFDILANIFSFLS---PDS------LARARSVCKNWHTCS--- 36
GmFBX187 --------------WSNLPLDILANIFSFLS---PDS------LARA-- 24
GmFBX60 -------IEKKKNLFSELTDDIVLNIFYKLED-DPRH------WARLACVCTKFSSLVRDFCWKTKCSLTIPQDLLSAAASDPPLSLHKLSFCCPGLRH-------------------------- 85
GmFBX7.1 --------------WANLPPELLLDIIRRVED-SETTWPARAVVVYCGSVCKSWRAVTKEIVKTPEQ-- 52
GmFBX270.1 -------------HWANLPPELLLDIIRRVED-SETTWPARAVVVYCGSVCKSWRAVTKEIVKTPEQ-- 53
GmFBX15.1 --------------WANLPSELLLDIIQRVEE-SETSWPARAVVVFCASVCKSWRSITREIVKTPEQ-- 52
GmFBX369 --------------WANLPSELLLDIIQRIEE-SETSWPARAVVVFCASVCKSWRSITREIVKTPEQ-- 52
GmFBX323.1 -------------RWANLPPELLLDIIQRLEA-SETSWPARRALVACASVCRLWREITKDVIKTP-- 51
GmFBX408.1 -------------RWANLPPELLLDIIQRLEA-SETSWPARRALVACASVCKLWREITKDVVKTPEQ-- 53
GmFBX20.1 -------------RWASLPPELLCDVIKRLEE-SESTWPARKHVVACAAVCKSWREMCKEIVTSPEF-- 53
GmFBX372.1 -------------RWASLPPELLRDVIKRLEE-SETTWPARKHVVACAAVCRSWRGMCKEIVTSPEF-- 53
GmFBX151 --------------WASLLLELLRDVINRLEA-SESTWPGCKHVVACAAMCKSWREMCKEIVSSPEF-- 52
GmFBX283 -------------RWASLPLELLGDVINRLEA-SESTWPGRKHVVACVAMCKSWREMCKEIVSSPEF-- 53
GmFBX262.1 -------------RWASLPPELLRDVINRLEA-SESTWPGRKHVVACAAVCKSWREMCKEIVSSPEF-- 53
GmFBX498.1 -------------RWASLPPELLRDVINRLEA-SESTWPGRKHVVACAAVCKSWREMCKEIVSSPEF-- 53
GmFBX181 -------------RWASLPPELLFDIIRRLEE-SENTWPARKHVVACAAVCQSWRNMCKDIVKSPEF-- 53
1.......10........20........30........40........50........60........70........80........90........100.......110.......120.....

```
GmFBX338     -------------RWASLPPELLFDIIRRLEE-SENTWPARKHVVACAAVCQSWRNMCKDIVKSPEF----------------------------------------------------------    53
GmFBX302     -------------CWANMPQELLREVLLRIEA-SEDTWPPRKSVVSCAGVCRSWRHITKDIVKTP------------------------------------------------------------    51
GmFBX345     -------------CWANMPQELLREVLFRIEA-SEDAWPPRKSVVACAGVCRSWRQITIDIVKT-------------------------------------------------------------    50
GmFBX29      -------------CWANMPPELLRDVLMRIEA-SEDSWPAQKHVVACAGVCRSWREIMKEI----------------------------------------------------------------    47
GmFBX160.1   ----------------NMPRELLREVLLRIES-SEATWPSRRSVVACGGVCRTWRLIVKEIV---------------------------------------------------------------    45
GmFBX386.1   -------------WWANMPHELLREVLLRIES-SESTWPLRRSVVACGGVCRTWRRL--------------------------------------------------------------------    43
GmFBX286     --------------WSSMLPEILGEIVRRVDA-AEEQWPNRQNVVACACVCKRWRDITREVV---------------------------------------------------------------    47
GmFBX312     --------------WSNMLPEILGEIVRRVDA-AEEQWPNRQNVVACACVCKRWRDITREVV---------------------------------------------------------------    47
GmFBX33.1    -------------DWSELTRECLINILSRLSV-EDR-------WRGTMLVCKSWFSVFKEPSLH-------------------------------------------------------------    43
GmFBX67      --------------WGELPPELLESISKTLTI-YVD-------YLRFRSVCRSWRSSVPKIPLH-------------------------------------------------------------    42
GmFBX307     -------------DWSNLPRDLVSQIVSGLGL-ID--------FLSFRGVCEDWRVA--------------------------------------------------------------------    35
GmFBX340     --------------WSNLPHDLLSRISSGLGL-ID--------FLSFRGVCKDWRVVS-------------------------------------------------------------------    35
GmFBX217.1   -------------DWSSLPRGILEMVAERLTF-ID--------CLSISKVCTSWNRV--------------------------------------------------------------------    35
GmFBX190     ------------EGIGKVPEDVVIEILVRGG--IREWE-------QISCVKKQWASLFRCDCECFWQAALSFYYPHQLPLPPPPTWPVPLPNNSKR-----------------------------    75
             1.......10........20........30........40........50........60........70........80........90.......100.......110.......120.....
```

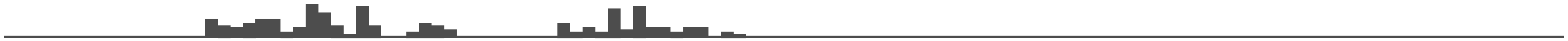

Supplement: Supplementary file 1 [file ijms-18-00818-s001.zip › ijmssupplementary/3S2file-F-box motifalignmentsis.pdf]
