# Supplementary figures and images for: Genome-Wide Analyses of the Soybean F-Box Gene Family in Response to Salt Stress
Source: Int J Mol Sci. 2017 Apr 12;18(4):818. doi: 10.3390/ijms18040818 (PMC5412402; doi:10.3390/ijms18040818)

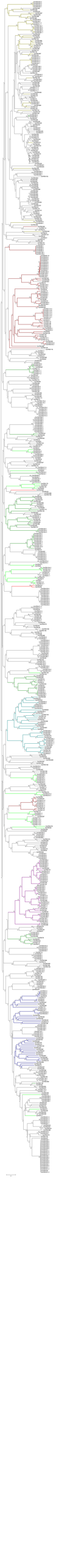

Supplement: Supplementary file 1 [file ijms-18-00818-s001.zip › ijmssupplementary/6FileS3-phylogenetictreeCterminiis.pdf]

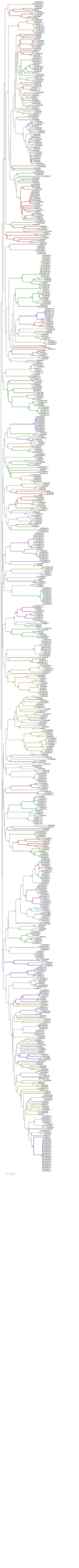

Supplement: Supplementary file 1 [file ijms-18-00818-s001.zip › ijmssupplementary/7FileS4-phylogenetictreeintronnumberis.pdf]

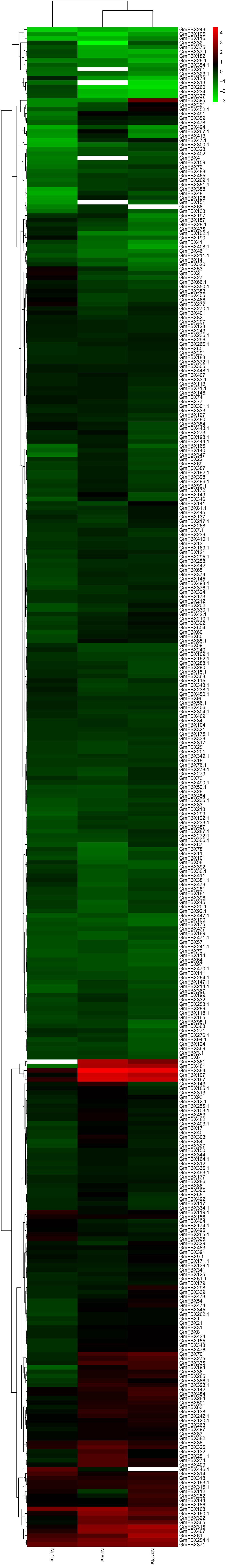

Supplement: Supplementary file 1 [file ijms-18-00818-s001.zip › ijmssupplementary/9FileS5-heatmap under salt stressgeohdzipis.pdf]
